# PEGylated Molybdenum–Iodine Nanocluster as a Promising Radiodynamic Agent against Prostatic Adenocarcinoma

**DOI:** 10.1021/acs.inorgchem.4c00084

**Published:** 2024-02-16

**Authors:** Tomáš Přibyl, Michaela Rumlová, Romana Mikyšková, Milan Reiniš, Antonín Kaňa, Karel Škoch, Jaroslav Zelenka, Kaplan Kirakci, Tomáš Ruml, Kamil Lang

**Affiliations:** † Department of Biochemistry and Microbiology, University of Chemistry and Technology Prague, 166 28 Praha 6, Czech Republic; ‡ Department of Biotechnology, University of Chemistry and Technology Prague, 166 28 Praha, Czech Republic; § Institute of Molecular Genetics of the Czech Academy of Sciences, Vídeňská 1084, 142 20 Praha, Czech Republic; ∥ Department of Analytical Chemistry, University of Chemistry and Technology Prague, 166 28 Praha, Czech Republic; ⊥ Institute of Inorganic Chemistry of the Czech Academy of Sciences, Řež 1001, 250 68 Husinec-Řež, Czech Republic

## Abstract

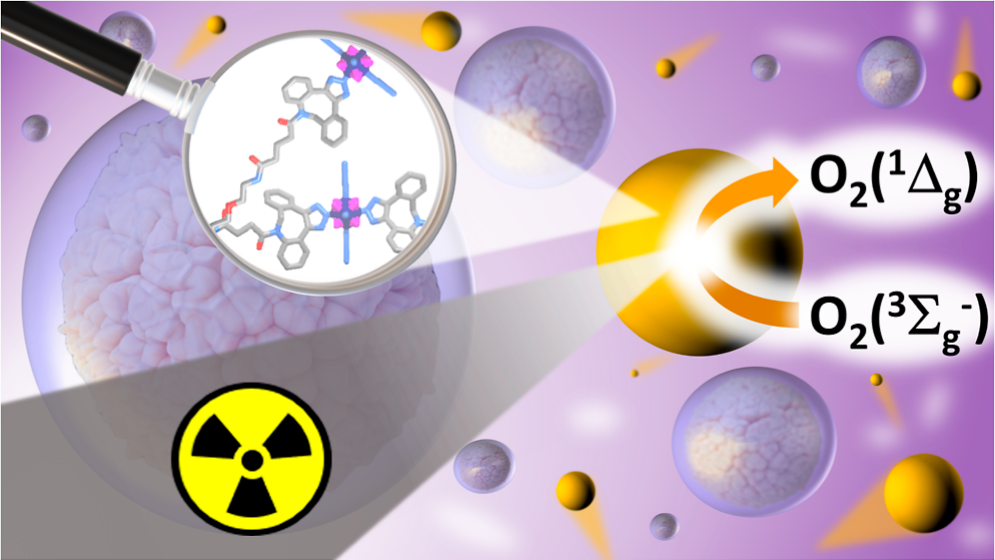

The combination of photodynamic therapy and radiotherapy has given rise to a modality called radiodynamic therapy (RDT), based on reactive oxygen species-producing radiosensitizers. The production of singlet oxygen, O_2_(^1^Δ_g_), by octahedral molybdenum (Mo_6_) clusters upon X-ray irradiation allows for simplification of the architecture of radiosensitizing systems. In this context, we prepared a radiosensitizing system using copper-free click chemistry between a Mo_6_ cluster bearing azido ligands and the homo-bifunctional linker bis-dPEG_11_-DBCO. The resulting compound formed nanoparticles, which featured production of O_2_(^1^Δ_g_) and efficient cellular uptake, leading to remarkable photo- and radiotoxic effects against the prostatic adenocarcinoma TRAMP-C2 cell line. Spheroids of TRAMP-C2 cells were also used for evaluation of toxicity and phototoxicity. In vivo experiments on a mouse model demonstrated that subcutaneous injection of the nanoparticles is a safe administration mode at a dose of up to 0.08 g kg^–1^. The reported results confirm the relevancy of Mo_6_-based radiosensitizing nanosystems for RDT.

## Abstract 2

The combination of photodynamic therapy and radiotherapy has given rise to a modality called radiodynamic therapy (RDT), based on reactive oxygen species-producing radiosensitizers.

## Introduction

Photodynamic therapy (PDT) is a minimally invasive modality for the treatment of several malignancies.^1^ Its principle lies in producing reactive oxygen species (ROS), mostly singlet oxygen, O_2_(^1^Δ_g_), by a photosensitizer upon visible light irradiation. Still, the conventional PDT treatment is limited to tumors located at the surface or a few millimeters under the skin or inside cavities because of the limited penetrability of visible light through tissues.^2^ To overcome this obstacle, several alternative ways of excitation have been employed such as infrared irradiation exciting two-photon absorbing photosensitizers,^3^ chemiluminescent nanoparticles able to excite photosensitizers in situ,^4^ or X-ray irradiation to activate ROS-producing radiosensitizers.^5^ The latter-named alternative of activation is probably the most promising because X-rays can reach deep-seated tumors. The clinical translation of this modality is facilitated by available equipment originally developed for radiotherapy treatment.^5^ Radiodynamic therapy (RDT) was first introduced in the form of a complex system composed of scintillating nanoparticles which transfer light energy to porphyrinic photosensitizers immobilized within the pores of a mesoporous silica shell covering the nanoscintillator.^6^ Since then, several other groups have reported promising results regarding the use of RDT against cancer.^7,8^ Recently, our group reported the production of O_2_(^1^Δ_g_) by octahedral molybdenum (Mo_6_) cluster complexes upon X-ray irradiation.^9^

These metallic aggregates surrounded by eight iodine inner ligands and six apical ligands form long-lived triplet states when excited by X-rays, electrons, or UV–visible light. Thus, the formed triplet states relax via red-NIR phosphorescence^10^ or energy transfer to molecular oxygen to form O_2_(^1^Δ_g_) with high quantum yields, making Mo_6_ clusters exclusively type II photosensitizers.^11,12^ These properties have led to various applications in PDT,^13−20^ photoinactivation of bacteria,^21−24^ and recent studies have shown the potential of these clusters for RDT.^25−28^ The direct administration of Mo_6_ clusters is complicated by their coordination instability in aqueous media, which leads to the displacement of apical ligands by water molecules, causing the formation of large aggregates, and worsening sensitizing and cellular uptake properties.^13^ The hydrolytic process can be mitigated via the attachment of bulky hydrophobic apical ligands; however, these clusters display poor water solubility, thus limiting their deposition at tumor sites.^14^ PEGylation has proven to be a successful strategy for the stabilization of therapeutic molecules and nanoparticles in an aqueous medium. It can significantly reduce the uptake by the reticuloendothelial system and increase their circulatory half-time, as well as providing high colloidal stability in a biological medium.^29^

**Figure 1:**
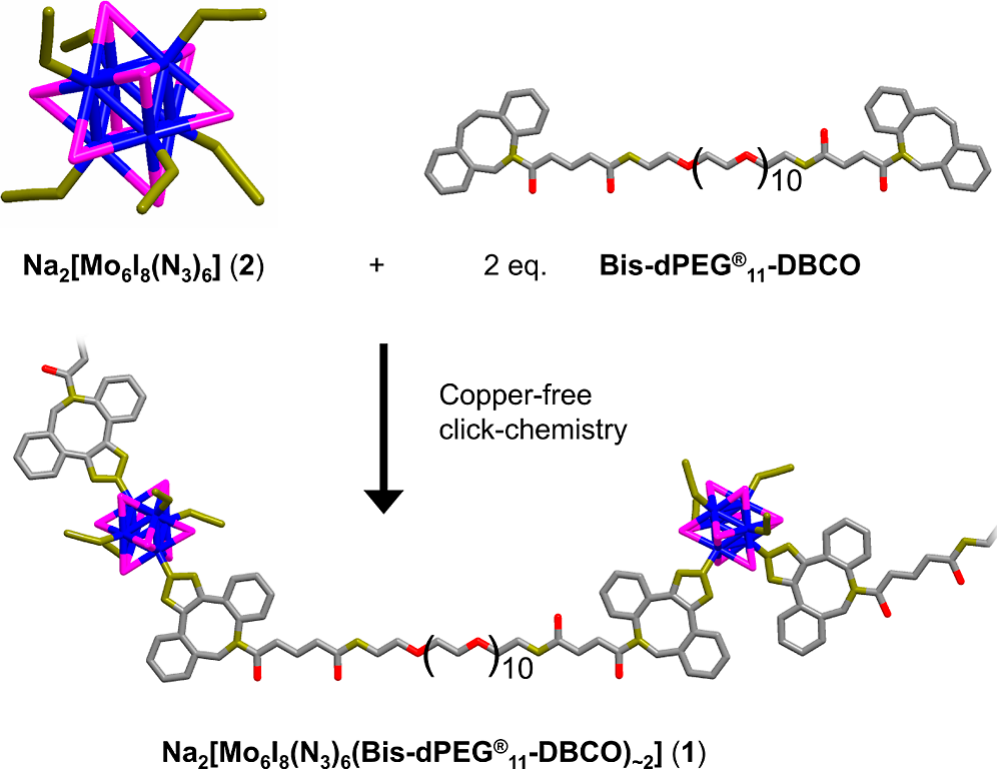
Schematic representation of the copper-free click chemistry reaction between Na_2_[Mo_6_I_8_(N_3_)_6_] (**2**) and the homo-bifunctional linker bis-dPEG_11_-DBCO, yielding Na_2_[Mo_6_I_8_(N_3_)_6_(bis-dPEG_11_-DBCO)_∼2_] (**1**). Color coding: molybdenum in blue, iodine in magenta, carbon in gray, nitrogen in dark yellow, and oxygen in red. Sodium counterions and hydrogen atoms have been omitted for clarity.

Herein, we designed a radiosensitizing system using copper-free click chemistry between a Mo_6_ cluster bearing azido ligands and the homo-bifunctional linker bis-dPEG_11_-DBCO (Figure 1). The composition was confirmed using ^1^H and ^13^C NMR and ICP–MS, the colloidal stability and zeta potentials of phosphate-buffered saline (PBS) dispersions were studied by dynamic light scattering, and the phosphorescence and photosensitizing activities were analyzed using luminescence spectroscopy. The biological activity of the nanoparticles in the context of PDT and RDT was evaluated against TRAMP-C2 cells and TRAMP-C2 spheroids, and the in vivo acute toxicity was studied in a mice model.

## Results and Discussion

### Preparation and Characterization

The PEGylated cluster (**1**) was prepared by copper-free click chemistry using a similar procedure as previously reported.^16^ In brief, Na_2_[Mo_6_I_8_(N_3_)_6_] (**2**) was allowed to react with 2 molar equivalents of the homo-bifunctional linker bis-dPEG_11_-DBCO in DMSO at room temperature for 4 days, and a purification procedure was carried out in order to remove unreacted cluster and linker molecules (see Experimental section). Note that a higher linker-to-cluster ratio led to extensive cross-linking of Mo_6_ clusters, resulting in the formation of insoluble aggregates. The reaction between **2** and the linker was monitored by ^1^H and ^13^C NMR and revealed the presence of approximately 7% of an unreacted DBCO fragment as estimated from ^1^H NMR, probably originating from molecular fragments where only half of the organic linker has reacted (Figure S1, Supporting Information). In obtained material **1**, acetylene peaks originally observed in ^13^C NMR spectra (δ_C_ = 108.2 and 114.3 ppm) were replaced by two additional downfield signals characteristic for aromatic carbons (δ_C_ = 125–145 ppm), indicating the formation of triazolate moieties. As previously reported, triazolate-containing Mo_6_ complexes show coordination of the central nitrogen atom of the triazolate heterocycle to molybdenum atoms of the cluster core.^30^

**Figure 2:**
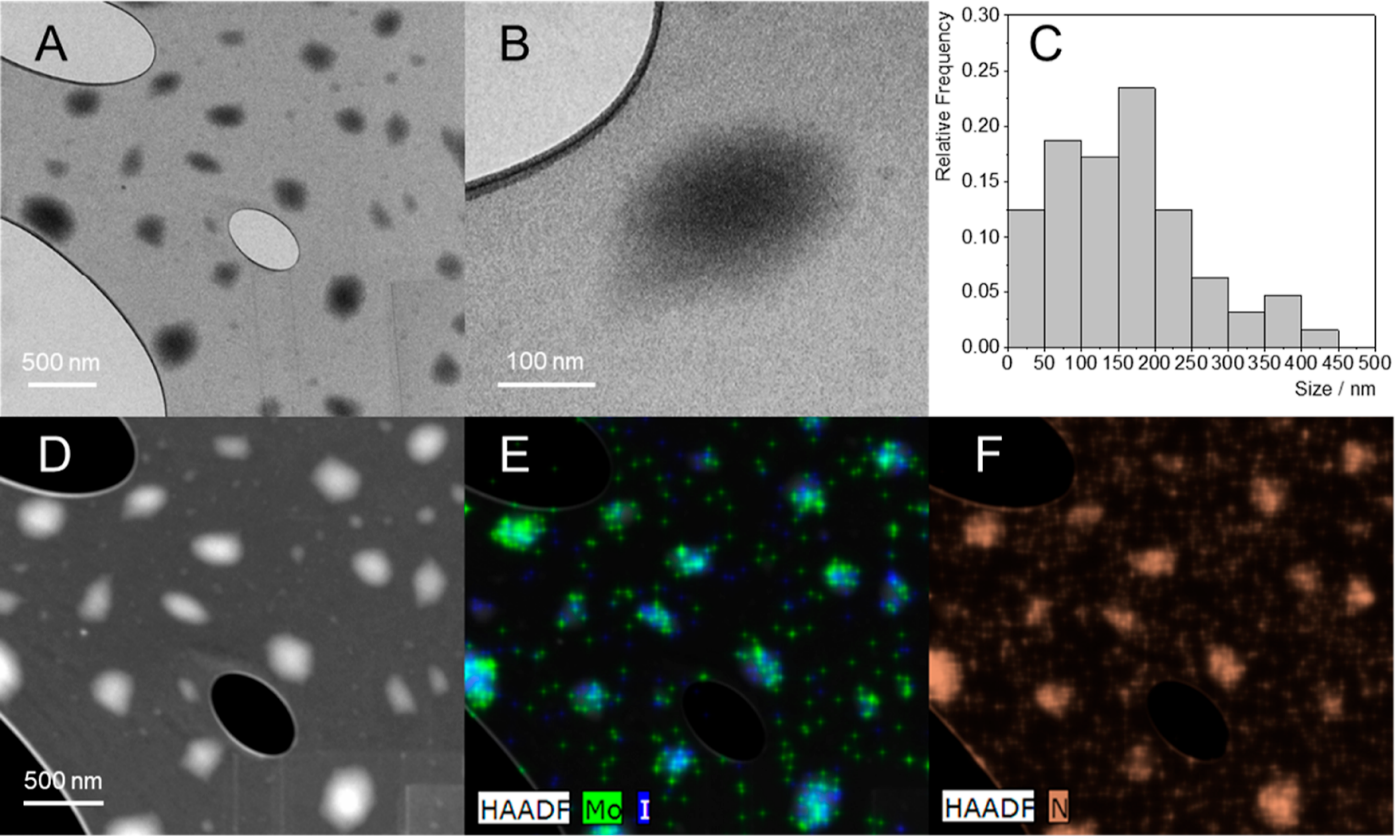
TEM images of **1** in the bright field (A, B) with the corresponding particle size distribution (C), and in the dark field (D) with the Mo, I (E), and N (F) HAADF elemental mapping.

The composition of **1** was evaluated by ICP–MS, evidencing a mass content of 13.75 wt % of molybdenum corresponding to 45.12 wt % of **2**. Thus, the approximate chemical formula of compound **1** is Na_2_[Mo_6_I_8_(N_3_)_6_(bis-dPEG_11_-DBCO)_∼2_]. TEM imaging of a water dispersion of **1** revealed nanoparticles with a mean size of 161 ± 92 nm, highlighting the high degree of cross-linking between the clusters. These nanoparticles were composed of Mo, I, and N as demonstrated by HAADF elemental mapping (Figure 2). Measurement of **1** (0.1 mg mL^–1^) in PBS by dynamic light scattering evidenced good colloidal stability of the nanoparticles with a mean size by number of 168 ± 54 nm (*Z*-average = 204 nm, PDI = 0.14) comparable to that observed by microscopy, and an average zeta potential of −8.9 ± 2.3 mV (Figure S2, Supporting Information). No significant changes in the size distribution and zeta potential were observed for the nanoparticles after 8 days in PBS, highlighting the colloidal stability in this medium (Figure S2, Supporting Information).

**Figure 3:**
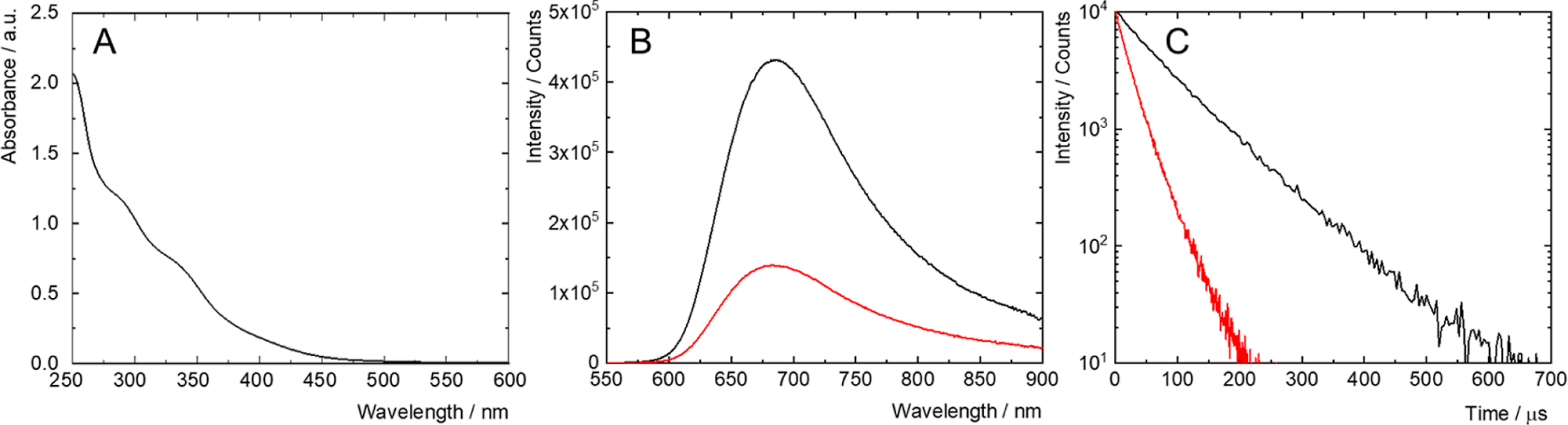
(A) Absorption spectra of **1** in PBS. (B) Phosphorescence emission spectra of **1** in air- (red) and argon- (black) saturated PBS, excited at 400 nm. (C) Phosphorescence decay kinetics at 700 nm of **1** in air- (red) and argon- (black) saturated PBS, excited at 405 nm.

The photophysical properties of **1** were studied in PBS at a concentration of 0.1 mg mL^–1^ and are summarized in Table 1. The absorption spectrum of a PBS dispersion of the nanoparticles was typical of Mo_6_ clusters with broad absorption bands in the UV/blue region and an onset at approximately 500 nm (Figure 3A). When excited at 400 nm, the dispersion displayed a broad red-NIR phosphorescence band originating from Mo_6_ clusters, with a maximum located at 686 nm, quantum yield of 0.21 ± 0.02, and lifetime of 77 μs in argon-saturated PBS (Figure 3B,C). Note that the phosphorescence decay curve could be fitted by a single exponential function, indicating homogeneous luminescence properties within the population of PEGylated clusters. A decrease in the phosphorescence quantum yield to 0.07 ± 0.01 and of the lifetime to 23 μs was observed in air-saturated PBS, indicating efficient quenching of the emissive triplet states by oxygen. The stability of the photophysical properties was evaluated by comparing photophysical parameters of fresh and 8 days old PBS dispersions of **1**. It revealed no significant changes in these parameters, highlighting the long-term stability in this medium (Figure S3, Supporting Information), as opposed to **2**, which was previously reported to undergo hydrolysis in aqueous medium.^31^

**Table 1:** Photophysical Properties of **1** in PBS at Room Temperature^a^

sample	λ_L_ (nm)	Φ_L_	Φ_air_	τ_L_ (μs)	τ_air_ (μs)
**1**, fresh	686	0.21 ± 0.02	0.07 ± 0.01	77	23^b^
**1**, 8 days in PBS	687	0.20 ± 0.02	0.07 ± 0.01	76	23^b^

a λ_L_—phosphorescence maximum (λ_exc_ = 400 nm); τ_L_ and τ_air_—phosphorescence lifetimes in oxygen-free PBS and amplitude average lifetimes in air-saturated PBS, respectively (λ_exc_ = 405 nm, λ_em_ = 700 nm); Φ_L_ and Φ_air_—phosphorescence quantum yields in oxygen-free and air-saturated PBS, respectively (λ_exc_ = 320–400 nm, experimental error of Φ_L_ is ±0.01).

b Biexponential decay.

**Figure 4:**
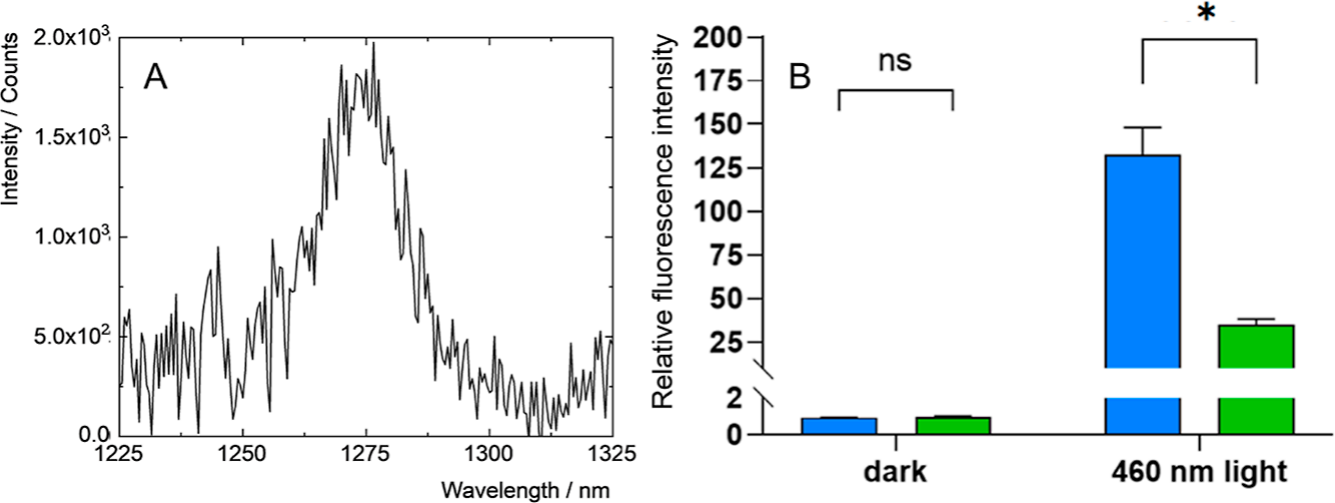
(A) Phosphorescence signal of O_2_(^1^Δ_g_) produced by **1** in oxygen-saturated PBS, excited at 400 nm. (B) Formation of ROS in the full medium kept in dark (left) or irradiated (1 min, 460 nm, right) probed with 10 μM DCF-DA in the presence of 90 μg mL^–1^**1** (blue bar) and the absence of **1** (the control, green bar); fluorescence intensity relative to the non-irradiated control, ns indicates nonsignificant signals, and * represents statistically significant differences according to the Student’s *t*-test (*p* < 0.05).

The efficient quenching of the triplet states by oxygen observed for the PBS dispersions of **1** suggested the production of O_2_(^1^Δ_g_). This feature was confirmed by measuring its NIR phosphorescence at 1274 nm (Figure 4A). While it was not possible to obtain the quantum yield of the production of O_2_(^1^Δ_g_) due to light scattering caused by the nanoparticles, the fraction of the formed excited states quenched by oxygen in the air atmosphere *P*_T_^O_2_^ (*P*_T_^O_2_^ = 1 – τ_air_/τ_L_) was equal to 0.70, suggesting an efficient production of O_2_(^1^Δ_g_).

As the basis of the PDT treatment is the induction of intracellular oxidative stress by photosensitized ROS, we further evaluated the formation of ROS using a 2′,7′-dichlorofluorescein diacetate (DCF-DA) probe in the full medium used for following in vitro biological experiments (Figure 4B). The illumination of the probe in the medium clearly led to the oxidation of DCF-DA, indicating that the O_2_(^1^Δ_g_) produced by the nanoparticles of **1** can oxidize substrates and should induce intracellular oxidative stress.

### Uptake and Cellular Localization

**Figure 5:**
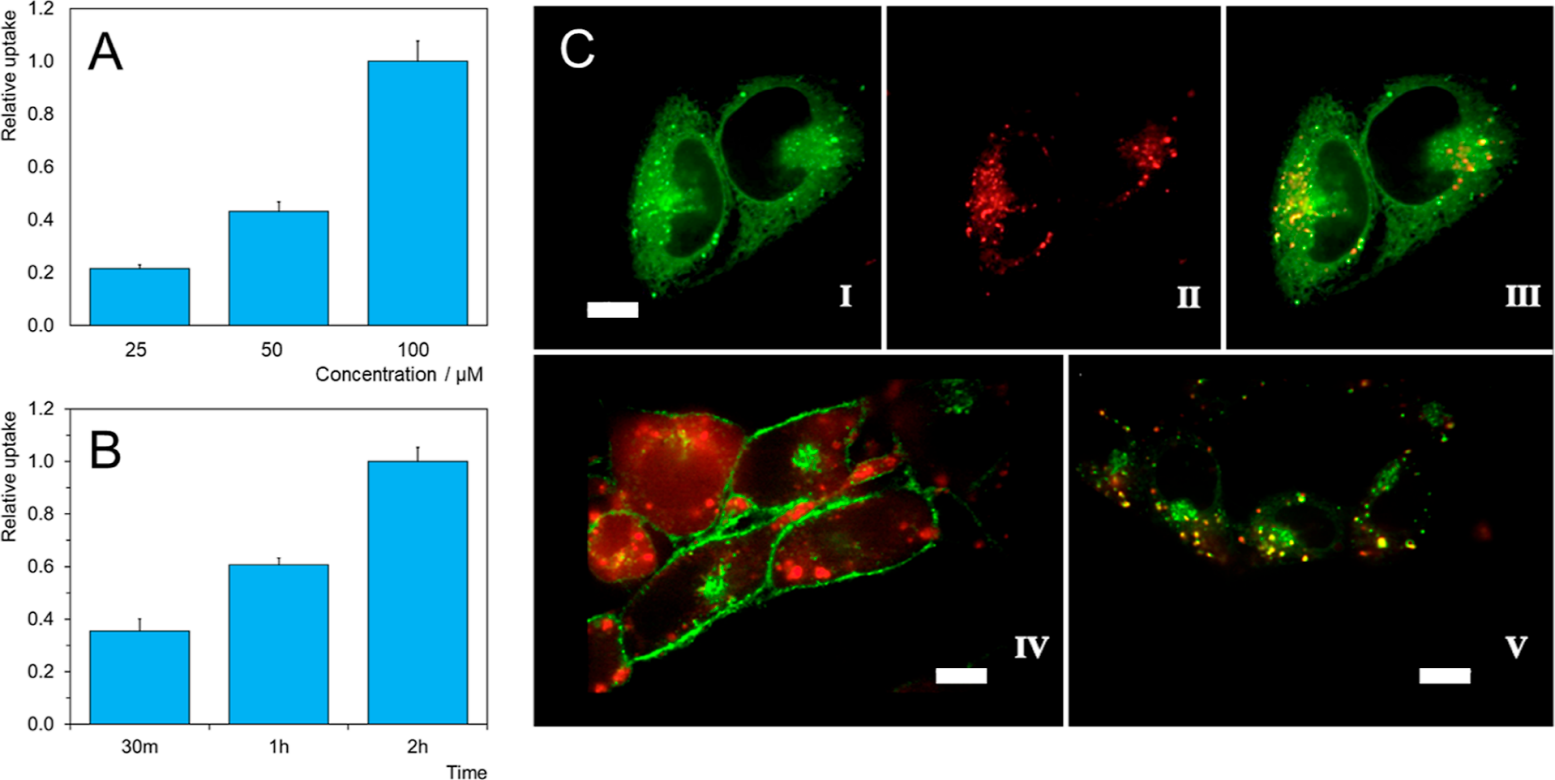
Uptake and localization of **1**: Uptake in TRAMP-C2 cells of (A) **1** in the concentration range of 0.1–0.4 mg mL^–1^, 2 h incubation. (B) **1** at a concentration of 0.4 mg mL^–1^, incubation time range 0.5–2 h. The results were normalized to the highest response. (C I−III) Colocalization of **1** in HeLa cells [I—lysosomes stained with LysoTracker Green (green), II—**1** (red), and III—merge], C IV—intracellular localization of **1** (red) in TRAMP-C2 cells with plasmatic membranes stained with WGA-FITC (green), and C V—colocalization of **1** (red) with lysosomes (green) in TRAMP-C2 cells. White bars represent 10 μm.

The biological activity of **1** was evaluated using prostatic adenocarcinoma cells TRAMP-C2. Indeed, prostate cancer remains one of the most prevalent and deadly cancer worldwide and is frequently treated with radiotherapy, and clinical studies also demonstrated the benefits of its treatment with PDT.^32,33^ Generally, a photo/radiosensitizer should be internalized or attached to cell membranes for the successful PDT/RDT treatment because the action radius of ROS is limited by their short lifetimes. Our previous work demonstrated low or no phototoxicity of photosensitizers, which are unable to enter cells or associate with their membranes.^13,16^ In the case of **1**, the flow cytometry measurements showed a linear dose- and time-dependent cellular uptake of **1** in TRAMP-C2 cells (Figure 5A,B), suggesting the good internalization of **1**.

The intracellular localization of **1** was investigated with spinning disc confocal fluorescence microscopy (Figure 5C). This technique demonstrated the presence of compound **1** in the cell cytoplasm. Since TRAMP-C2 cells bear a relatively smaller cytoplasm volume when compared to other cell lines, the lysosomal localization of **1** was first confirmed with HeLa cells with a large volume of the well-visible cytoplasm. Next, precise analysis of colocalization in TRAMP-C2 cells using a specific fluorescent probe, LysoTracker Green, revealed that **1** is readily sequestered by lysosomes, which agrees well with previously reported results on colocalizations of some Mo_6_-based photosensitizers.^14,15^ Lysosomes can safely store even the indigestible material while their damage upon irradiation releases proteases, which trigger apoptosis, a regulated and safe form of cell death.^34^

### Toxicity and Phototoxicity

**Figure 6:**
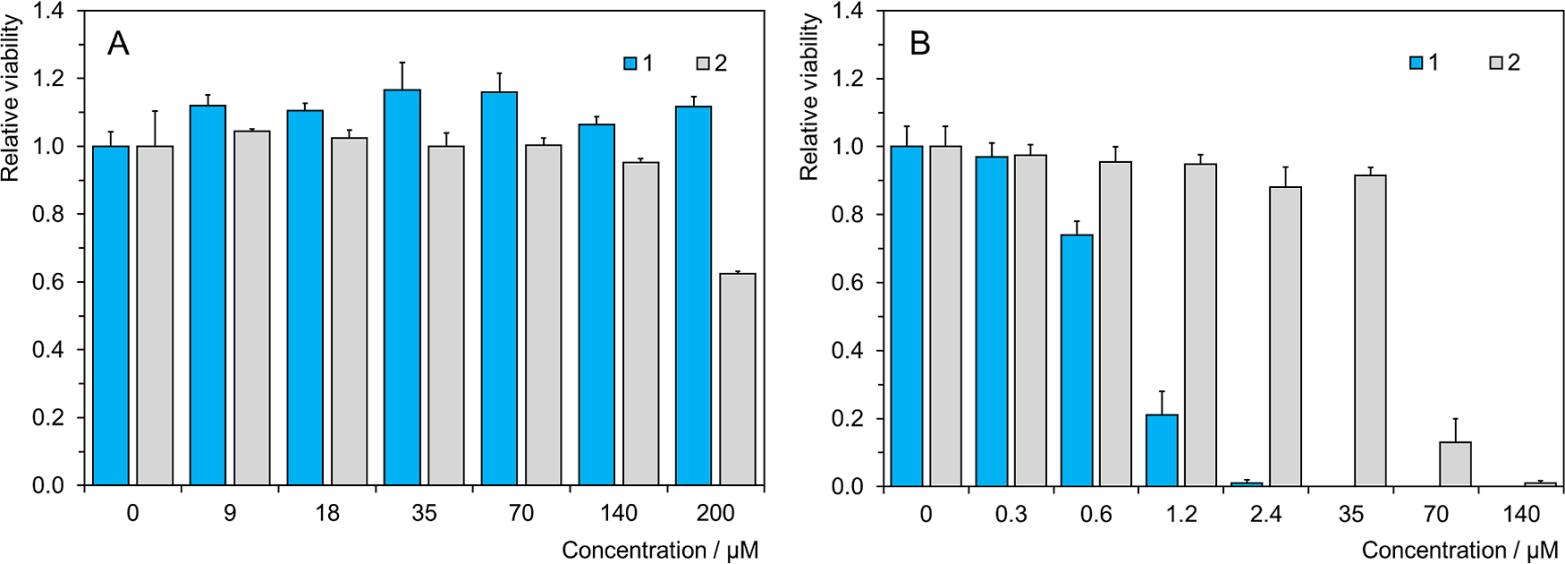
Toxicity toward TRAMP-C2 cells in the full media with indicated concentrations of **1** and **2**: (A) dark toxicity, 2 h loading, measured 24 h after incubation. (B) Phototoxicity, 2 h loading, illuminated with 460 nm light (18 mW cm^–2^, 15 min), viability measured 24 h after illumination.

No signs of dark toxicity toward TRAMP-C2 cells were observed in the presence of **1** at a concentration as high as 1.3 mg mL^–1^ ([Mo_6_] ∼ 300 μM), while its precursor, pure azido-cluster **2**, showed cytotoxic effects with an IC_50_ of 0.41 ± 0.01 μg mL^–1^ (220 ± 5 μM) (Figure 6A). On the other hand, **1** showed a high phototoxic effect after illumination with 460 nm light with an IC_50_ of 3.2 ± 0.1 μg mL^–1^ (0.75 ± 0.02 μM in Mo_6_), while the phototoxic effect of **2** was approximately 2 orders of magnitude worse with an IC_50_ of 102 ± 4 μg mL^–1^ (54 ± 2 μM) (Figure 6B). Clearly, connecting **2** via the linkers not only led to a decrease in inherent dark toxicity but also severely increased phototoxicity of the nanoparticles made of **2**. Even more, the photodynamic effect of **1** surpassed that of common photosensitizers such as Foscan (IC_50_ = 1.8 μM) or typical chemotherapy agents such as cisplatin (IC_50_ = 2.4 μM) used for prostate cancer therapy.^35,36^

The phototoxicity of **1** is comparable to that of the most efficient Mo_6_-based nanosystems, such as cluster-loaded PLGA nanoparticles.^19,37^ However, in contrast to these systems, **1** had no dark toxicity at high Mo_6_ concentrations. Several molecular Mo_6_ photosensitizers showed even lower IC_50_ values for phototoxicity. In these cases, very low water solubilities disqualify them from in vivo experiments.^13,16^

### Formation of ROS

**Figure 7:**
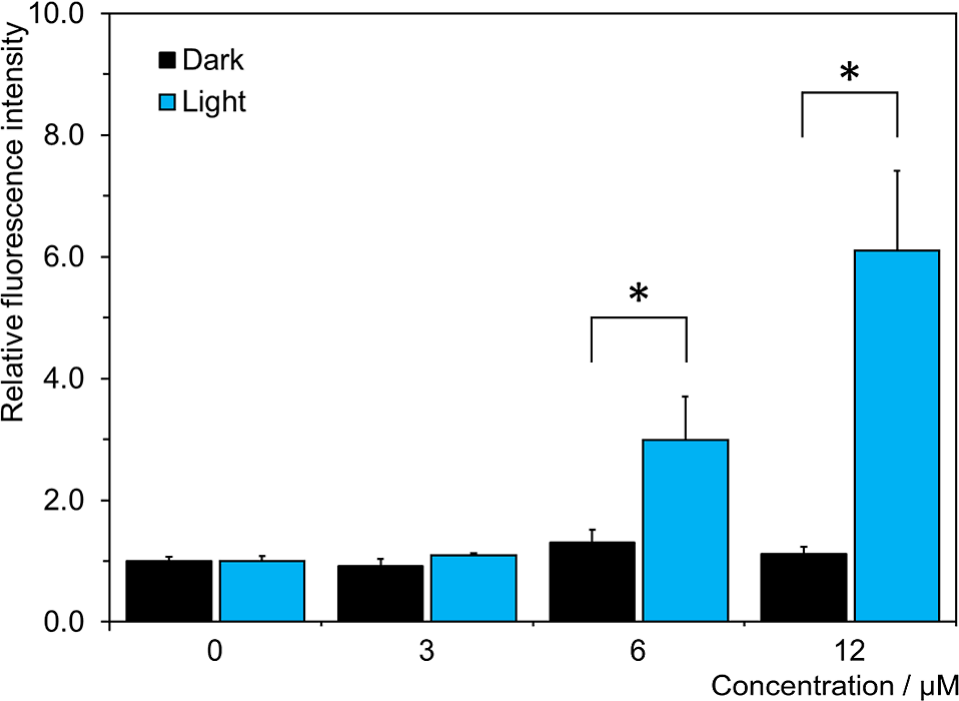
Formation of ROS in TRAMP-C2 cells incubated for 2 h with indicated concentrations of **1**, then left in the dark (black) or illuminated with 460 nm light for 15 min (blue), and finally treated with 10 μM DCF-DA for 30 min. * represents statistically significant differences according to the Student’s *t*-test (*p* < 0.05).

The primary purpose of a photosensitizer in PDT is to increase the production of ROS, including O_2_(^1^Δ_g_), in the cellular environment, to impose oxidative stress and finally to induce cell death, resulting in the eradication of tumor tissues.^38^ However, cancer cells resist oxidative stress and programed cell death, complicating the original simple premise.^39^ We have already shown above that **1** photo-oxidizes DCF-DA, an ROS probe, in dispersion. In this line, the measure of oxidative stress imposed by **1** in TRAMP-C2 cells was also investigated. Cells were treated with **1** and irradiated with 460 nm light, and then the post-PDT concentrations of ROS in cells were evaluated upon the addition of DCF-DA and careful removal of the medium. The results showed an increased formation of ROS with an increased concentration of **1** in illuminated cells, but not in the dark (Figure 7). It indicates oxidative stress following the PDT treatment, in line with the phototoxic effects reported above.

### Cell Death Mode

**Figure 8:**
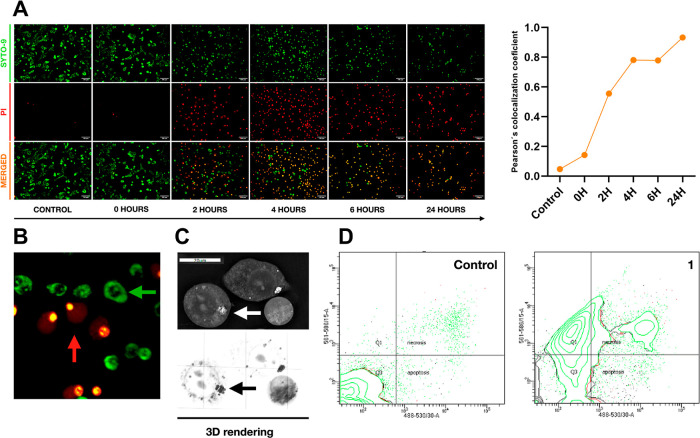
Cell death mode of TRAMP-C2 cells after the PDT treatment with **1**: (A) PI and SYTO-9 staining (white bar represents 100 μm) (left) and changes in Pearson’s colocalization coefficient over time (right). (B) Zoomed swelling of cells, SYTO-9 positive cells (green arrow), and SYTO-9 and PI positive cells (red arrow). (C) Holotomographic reconstruction of morphological changes in detail (white bar represents 20 μm) and lipid droplet aggregation (black, white arrow). (D) Flow cytometry analysis of cells stained with annexin-V and PI.

In the presence of ROS, cells can undergo conventional programed death (apoptosis) or necrosis. In recent years, nonconventional modes of cell death possibly induced by PDT, such as paraptosis, necroptosis, ferroptosis, etc., have been described.^40^ After PDT treatment with **1**, cells were not permeable to propidium iodide (PI), which is typically impermeable to living cells. Over time, the intensity of the signal of PI-positive cells increased (Figure 8A), which indicated the progress of cell decease. The dying cells exhibited specific morphological changes, such as cell swelling and rounding (Figure 8B,C). Additionally, the mitochondrial network fragmented (Figure S4) and lipid droplets aggregated (Figures 8C and S4). Staining with PI and annexin-V, and flow cytometry, commonly used for the apoptotic cell death assay, revealed that only 4% of cells are considered apoptotic (16% necrotic), compared to 2% (6% necrotic) in the nontreated control cell group. Nevertheless, more than 50% of the cell population after PDT treatment moved to the Q1 area in the fluorescence-activated cell sorting (FACS) diagram (Figure 8D), suggesting the occurrence of ferroptosis.^41^ Ferroptosis is a nonapoptotic peroxidation-driven cell death mode, which is iron-dependent, and the cells undergo lipid peroxidation caused by ROS. During this mode cells are rounding and swelling due to the exchange of Ca^2+^ ions and water between the cell and environment.^40^ Thus, Mo_6_-based photo/radiosensitizers can act as ferroptosis inducers. Further research is needed to explore this aspect in more details.

### Spheroid Toxicity and Phototoxicity

**Figure 9:**
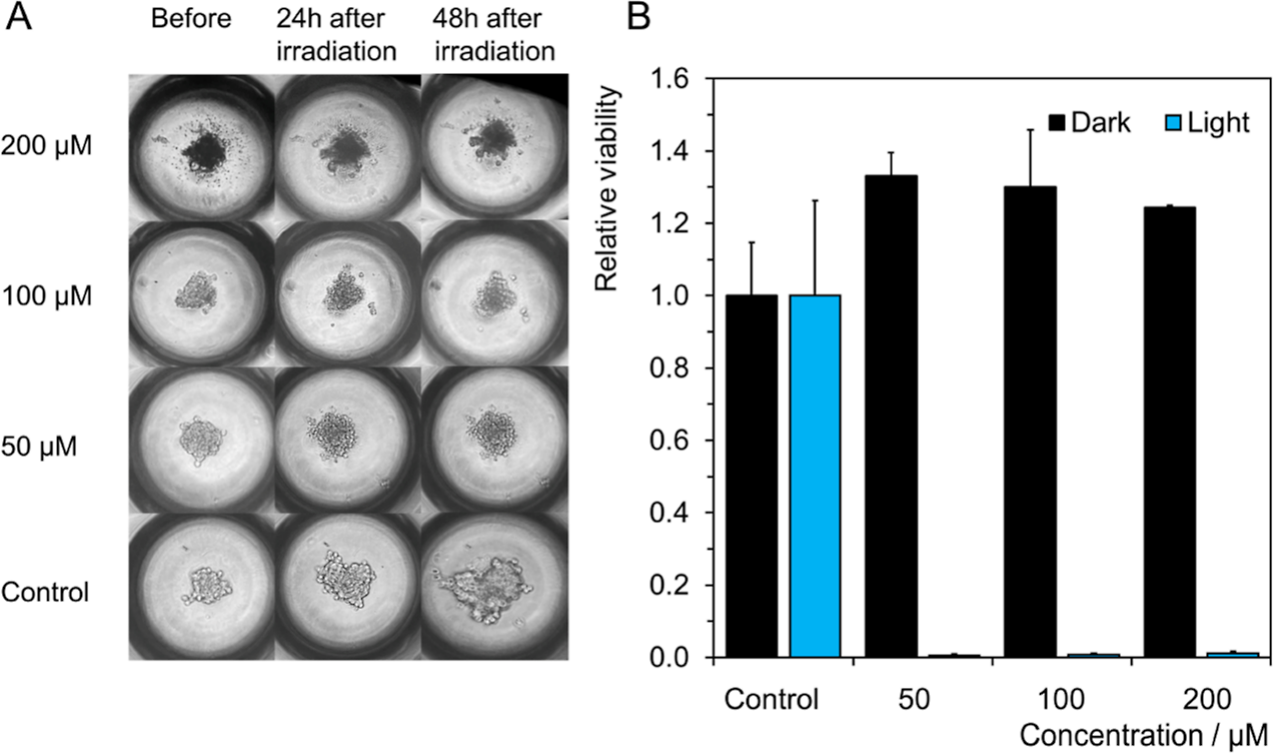
(A) Microscopic images of 24 h-old spheroids formed by TRAMP-C2 cells together with indicated concentrations of **1** taken before irradiation and 24 h or 48 h after irradiation with 460 nm light (18 mW cm^–2^, 15 min). (B) Toxicity of indicated concentrations of **1** toward 24 h-old TRAMP-C2 spheroids kept in the dark (black) or after irradiation with 460 nm light (18 mW cm^–2^, 15 min) (blue) measured after 48 h as the cell viability using the resazurin metabolic test.

Three-dimensional cultures represent suitable in vitro models of in vivo hypoxia, lactic acidosis, and cell–cell interactions, helping to comprehensively improve knowledge of clinical applicability before translating the research to in vivo studies.^42^ Our previous work demonstrated that the PDT effect of Mo_6_ clusters is significantly modified by lactic acidosis (increased sensitivity of cells) and hypoxia (decreased efficiency of PDT), which is relevant to in vivo conditions.^27^ Therefore, the spheroids of TRAMP-C2 cells were grown for 24 h in the presence of **1**, ranging from 50 to 200 μM (in Mo_6_), then left in the dark or irradiated with 460 nm light. No dark toxicity was observed at these concentrations, while a growth arrest and loss of metabolic activity of the spheroids were evidenced after irradiation (Figure 9). These results suggest the potential of **1** to deactivate cancer cells under the more relevant conditions.

### Radiotoxicity

**Figure 10:**
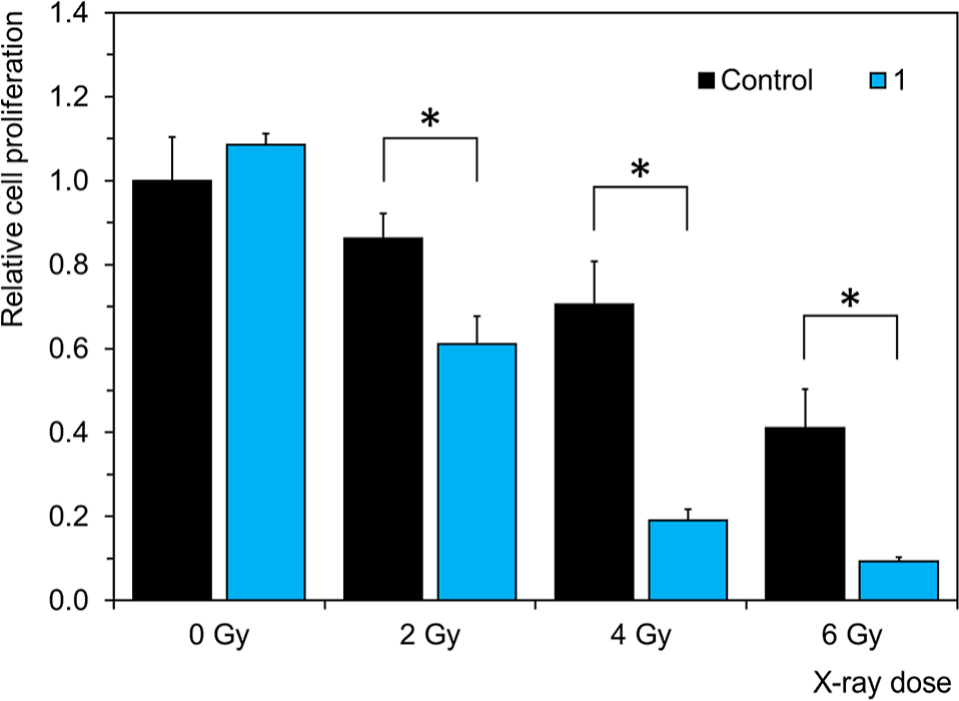
Radiotoxicity of **1** at a concentration of 1.3 mg mL^–1^ toward TRAMP-C2 cells incubated for 2 h and irradiated with indicated X-ray doses. Control bars represent the same experiments performed in the absence of **1**.

Since our previous studies on Mo_6_ clusters demonstrated their synergistic effects on cell proliferation arrest upon X-ray irradiation,^25−27^ we investigated the potential of **1** as an RDT sensitizer. For the radiotoxicity evaluation, TRAMP-C2 cells were incubated with **1** for 2 h at a nontoxic concentration of 1.3 mg mL^–1^ and irradiated with an equivalent of typical radiotherapeutic X-ray doses (2, 4, or 6 Gy). Cells were then seeded at 5% confluence, and their proliferation was determined after 72 h. A noticeable increase in the radiotoxic effect was observed for the cells incubated with **1**. Under these conditions, the dose-enhancement factor reached approximately 1.9, leading to a significant decrease in the irradiation dose necessary to achieve the desired antiproliferative effect (Figure 10). While the magnitude of the radiotoxic effect was similar^27^ or significantly better^25,26^ compared to the already published Mo_6_ materials, the benefit of the presented nanoparticles of **1** lies in the possibility to modify them by tumor-targeting groups (peptides or aptamers) in a simple click reaction exploiting the remaining unreacted azido ligands.

The radiosensitizing effect of **1** is not as pronounced as its phototoxicity, and, therefore, higher concentrations are needed. This feature is due to the weaker ability of X-rays to produce triplet states of Mo_6_ when compared with that of UV/visible light. However, the deeper penetration of X-rays over UV/visible light in tissues allows for the treatment of solid tumors unreachable by light. Also, radiotherapy is a widespread modality; thus, the clinical application of RDT could be facilitated by the pre-existing infrastructure. Note that the mechanisms at play in RDT and PDT are different. Mo_6_ clusters are type II photosensitizers, so they can be excited by UV/visible light to the excited singlet states followed by intersystem crossing to the triplet states, which in turn transfer energy to surrounding molecular oxygen to produce O_2_(^1^Δ_g_) in high quantum yields. X-rays do not only induce the formation of O_2_(^1^Δ_g_) via the limited formation of the triplet states of Mo_6_ but also generate electrons (e.g., photoelectrons, Auger electrons, and secondary electrons) due to the presence of high-Z atoms in **1** and interaction with water molecules. These energetic electrons diffuse through tissues, interact with biomolecules and water molecules, and locally produce increased concentrations of free radicals including ROS.

### In Vivo Toxicity

**Figure 11:**
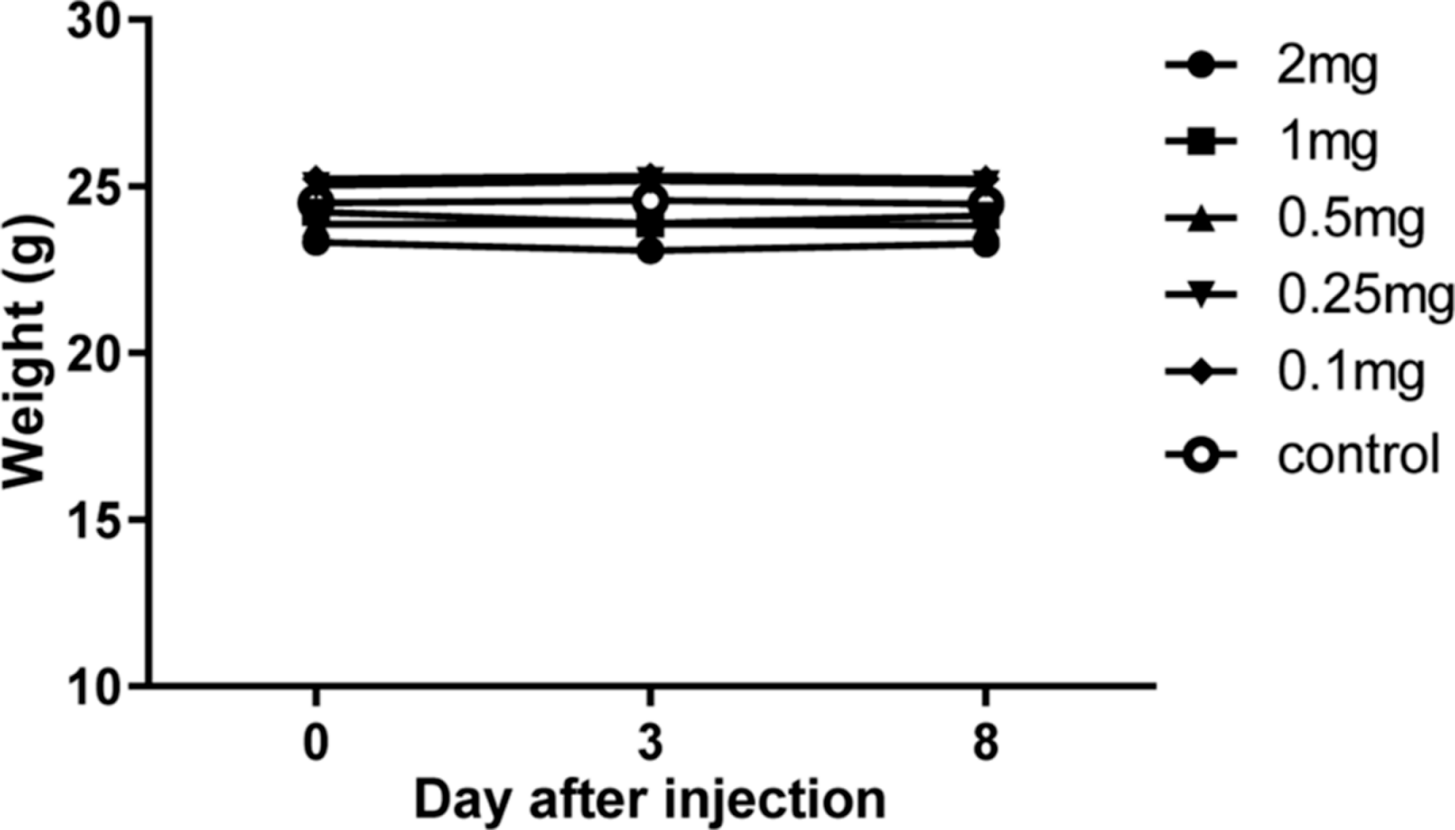
Effects of **1** in vivo: weight of mice after subcutaneous injection of **1**. Control mice were injected with a physiological saline solution only.

To analyze the toxic effects in vivo, B6 male mice were injected subcutaneously with **1**. No signs of whole or organ toxicity and no changes in body weight were observed for any amount of **1** used in the range 0.1–2 mg per mouse (4–80 mg kg^–1^), 8 days after injection compared to the control group (Figure 11). No significant changes in the blood count were found in the groups treated with **1**, compared with the control. All parameters were within the range of expected values for B6 mice (Table S1, Supporting Information). Splenocytes were analyzed by flow cytometry for a percentage of important selected immune cell populations CD4, CD8, CD11b+/Gr-1+ (myeloid-derived suppressor cells), and activated CD8+ immune cells (CD69+). Nanoparticles **1** did not affect the percentage of the basic immune cell population and did not affect CD8+ cell activation compared to that of untreated controls (Table S2, Supporting Information). Molybdenum content at the injection site and in the selected tissues determined by ICP–MS showed a dramatic increase in all the analyzed organs of the treated animals compared to controls. The highest accumulation, documented also by the yellow staining (Figure S5, Supporting Information), was consistently observed at the injection site. Lower values were observed in the liver, gallbladder, and kidney, the organs responsible for the detoxication. The molybdenum levels in phagocyte-rich organs, lungs, and spleen were lower, but orders of magnitude higher than in controls, suggesting relatively high mobility of **1** in the animal organism (Table S3, Supporting Information). No observed toxicity, the fact that both Mo and I are biogenic elements, high stability of the cluster core, and the presence of high amounts of Mo in the sites of Mo excretion (kidney and gallbladder) indicate that **1** is well tolerated even at such extreme doses and may not express any toxicity in the long term.^43^ This conclusion is in line with a finding that other octahedral transition metal clusters, made of non-biogenic elements (Re and Te), displayed toxicity at an order of magnitude higher than those employed in our study.^44^ We also previously demonstrated no toxicity of Mo_6_-based nanoparticles to mice and the occurrence of an increased Mo content in liver and kidney tissues. In that study, however, we employed different ligands with no ability to be further modified, and, most importantly, the resulting nanoparticles were not stable in biological medium, and, as a result, the total applied doses were lower.^27^

## Materials and Methods

### Reagents and General Procedures

Compound Na_2_[Mo_6_I_8_(N_3_)_6_] (**2**) was prepared according to a previously published procedure.^31^ Molybdenum, iodine, sodium azide, bis-dPEG_11_-DBCO (DBCO = dibenzocyclooctyne), dimethyl sulfoxide (anhydrous) (DMSO), and phosphate-buffered saline (10× concentrate, BioPerformance Certified) were obtained from Sigma-Aldrich and used as received.

### Preparation of **1**

Bis-dPEG_11_-DBCO (50 mg, 44 μmol) was dissolved into 2 mL of DMSO, and the resulting solution was added dropwise to 2 mL of a DMSO solution of Na_2_[Mo_6_I_8_(N_3_)_6_] (42 mg, 22 μmol) under magnetic stirring. The reaction mixture was left to stir at room temperature for 4 days, and then 45 mL of diethyl ether was added to trigger precipitation. The orange solid was dissolved in 2 mL of dichloromethane, 45 mL of acetone was added, and the orange solid was separated by centrifugation (10,000 rpm/5 min). This procedure was repeated, and the orange solid was washed twice with 40 mL of diethyl ether and dried under reduced pressure.

### Instrumental Techniques

NMR spectra were recorded on a JEOL Delta spectrometer (JEOL, Japan) (^1^H 600 MHz and ^13^C{^1^H} 151 MHz). Chemical shifts are given relative to tetramethylsilane (TMS) and referenced to the residual solvent signal (*d*_6_-DMSO: ^1^H 2.50 ppm; ^13^C 39.5). NMR assignments were supported by additional 2D-NMR experiments (gCOSY, HSQC, and HMBC). Images of the nanoparticles were acquired with a FEI Talos transmission electron microscope (Thermo Fisher Scientific, USA). The molybdenum content was determined by inductively coupled plasma mass spectrometry (ICP–MS, PerkinElmer, Concord, ON, Canada). The size distribution and zeta potentials in PBS were determined by dynamic light scattering on a particle size analyzer, Zetasizer Nano ZS (Malvern, UK). UV–vis absorption spectra of the solutions were recorded on a PerkinElmer Lambda 35 spectrometer. Phosphorescence properties were analyzed on an FLS1000 spectrometer (Edinburgh Instruments, UK) using a cooled PMT-980 photon detection module (Edinburgh Instruments, UK). Singlet oxygen phosphorescence was measured on a Fluorolog 3 spectrometer (Horiba Jobin Yvon, UK) using a Hamamatsu H10330-45 photomultiplier (Hamamatsu, Japan). The FLS1000 spectrometer was also used for time-resolved phosphorescence measurements (λ_ex_ = 405 nm, VPLED Series), and the recorded decay curves were fitted to exponential functions by the Fluoracle software (v. 2.13.2, Edinburgh Instruments, UK). Phosphorescence quantum yields were recorded using a Quantaurus QY C11347-1 spectrometer (Hamamatsu, Japan). Dispersions were saturated with air or argon to ensure different oxygen concentrations for phosphorescence analyses.

### Toxicity, Phototoxicity, and Radiotoxicity

Mouse prostatic adenocarcinoma cells (TRAMP-C2) were cultured in Dulbecco’s modified Eagle’s medium (DMEM medium, Sigma-Aldrich) supplemented with 5% FBS, 5% Nu-serum (Corning), 2 μg mL^−1^ insulin, and 10 mM *trans*-dehydro-androsterone (the full medium) at 37 °C in the atmosphere containing 5% of CO_2_. Experiments were performed 24 h after seeding. First, the full medium was exchanged for the full medium, which was phenol red-free. The clusters were dissolved in water, diluted to the required concentration and added into the full medium (phenol red-free) with cells. After 2 h, cells were irradiated or kept in the dark. Irradiation was performed with a 12 × 10 W LED source (Cameo) placed 25 cm from cells at 460 nm for 15 min (18 mW cm^–2^, in all cases below) or an X-RAD 225XL X-ray source (Precision X-RAY, Inc.) with an upper energy limit of 225 keV calibrated using a mouse phantom to determine the equivalent irradiation dose. The resazurin assay was used for the cell viability analysis after 24 h (phototoxicity) or cell proliferation when the cells were 10 times diluted and incubated for 72 h. In control experiments, the experimental conditions were the same; however, cells were treated only with the medium without **1**.

### Confocal Microscopy

TRAMP-C2 or HeLa cells were seeded into a 96-well plate with a glass bottom (Cellvis). The next day, cells were treated with **1** in the fresh full medium (phenol red-free) for 2 h and then washed and stained with LysoTracker Green (Thermo Fisher Scientific) or Wheat germ agglutinin-FITC conjugate (WGA-FITC, Thermo Fisher Scientific). A spinning disc confocal microscope (Revolution XD, Andor) was used with an excitation wavelength of 405 nm for monitoring **1** (emission 700 nm) or 488 nm for monitoring lysosomes and cytoplasmatic membranes (both emission at 525 nm). To observe the intracellular changes in the cell structure after exposure to light, cells were first seeded in MatTek glass bottom dishes. The following day, cells were treated with a solution containing **1** and fresh phenol-red full media and then incubated for 2 h. Then, they were illuminated at 460 nm (18 mW cm^–2^) for 15 min. Four hours after the illumination, cells were stained with DAPI (Thermo Fisher Scientific) and another dye, TMRE (Thermo Fisher Scientific), Nile Red (Thermo Fisher Scientific), or Lysotracker Green (Thermo Fisher Scientific). Finally, cells were examined under the confocal microscope.

### Cell Death Mode

To conduct live/dead staining, we first seeded TRAMP-C2 cells in MatTek glass bottom dishes. Next day, cells were incubated with **1** in fresh full medium, phenol red-free, for 2 h and illuminated for 15 min at 460 nm (18 mW cm^–2^). Then, the medium was exchanged for the fresh one, and cells were stained with propidium iodide (PI) and SYTO-9 Green Fluorescent Nucleic Acid Stain (both Thermo Fisher Scientific) at different times after illumination (up to 24 h) and analyzed using confocal microscopy. Alternatively, cells were not stained with PI or SYTO-9, and holotomographic microscopy (Nanolive) was performed. To conduct PI/annexin-V staining and flow cytometry analysis, cells were seeded in a 12-well plate. Next day, cells were incubated with **1** for 2 h in the full medium (phenol red-free) and illuminated at 460 nm (15 min). After the next 4 h, the wells were washed with PBS, trypsinized, again washed with PBS, stained with PI/annexin-V according to the manufacturer’s protocol for the Dead Cell Apoptosis Kit (Invitrogen) and analyzed by flow cytometry (BD FACSaria III).

### Spheroids

Spheroids of TRAMP-C2 cells were grown on a 96-well Ultra-Low Attachment (ULA) surface (Corning). The trypsinized suspension of cells was added to the wells with indicated concentrations of **1** (50, 100, and 200 μM in Mo_6_). After 24 h of incubation in a CO_2_ chamber, the full medium was exchanged for a fresh one (phenol red-free) and illuminated for 15 min with 460 nm light (18 mW cm^–2^). After 24 or 48 h, the viability was measured by using the resazurin assay.

### Uptake of **1**

TRAMP-C2 cells were seeded into 12-well plates. After 24 h, cells were treated with indicated concentrations of **1** for indicated times, then washed with PBS, trypsinized, and analyzed by flow cytometry (BD FACSaria III). The excitation wavelength was set to 405 nm, and emission was recorded within 655–685 nm.

### ROS Level in Medium

A 96-well plate was filled with the full medium (phenol red-free) and mixed with **1**. DCF-DA (10 μM) was added, and fluorescence was immediately measured. The plates were irradiated for 1 min at 460 nm, and then corresponding fluorescence emissions were measured. Excitation/emission wavelengths were 488/525 nm.

### ROS Level in Cells

TRAMP-C2 cells were seeded in a 96-well plate in full medium. The next day, the medium was replaced with the fresh full medium (phenol red-free) and incubated for 2 h with **1** (0, 3, 6, and 12 μM). After that, cells were irradiated with 460 nm light for 15 min (18 mW cm^–2^) or kept in the dark; DCF-DA (10 μM) was added, and the plates were kept in an incubator for 30 min. Then, the medium was carefully aspirated, and the fluorescence emissions were measured. Excitation/emission wavelengths were 488/525 nm.

### Mice

C57BL/6 (B6) male mice, 6–8 weeks old, were obtained from AnLab Co., Praha, Czech Republic. Experimental protocols were approved by the Institutional Animal Care Committee of the Institute of Molecular Genetics of the Czech Academy of Sciences, Praha. See the Supporting Information for more details.

## Conclusions

We have prepared an Mo_6_ cluster compound using a copper-free click chemistry reaction between Na_2_[Mo_6_I_8_(N_3_)_6_] and the homo-bifunctional ligand bis-dPEG_11_-DBCO. The resulting compound Na_2_[Mo_6_I_8_(N_3_)_6_(bis-dPEG_11_-DBCO)_2_] formed slightly negatively charged nanoparticles of approximately 160 nm in diameter. The nanoparticles displayed intensive red phosphorescence, efficiently quenched by oxygen, producing O_2_(^1^Δ_g_). The photophysical properties were unchanged over a week in PBS, highlighting the long-term stability of the nanoparticles in this physiological medium. The biological activity of the compound was tested in TRAMP-C2 prostatic adenocarcinoma cells. The compound internalized into lysosomes, displayed low dark toxicity and an intensive phototoxic effect upon 460 nm irradiation, even in the presence of fetal bovine serum. This feature was in line with the posttreatment intracellular oxidative stress evidenced with the help of the DCF-DA fluorescent probe. Similarly, the compound displayed strong phototoxicity and low dark toxicity against TRAMP-C2 cell spheroids. The combination significantly enhanced the radiotoxic effects of X-rays against TRAMP-C2 cells with a dose enhancement factor of approximately 1.9, resulting in a lower dose required to achieve the desired antiproliferative effect. Finally, the compound was tested in vivo in B6 mice, and its subcutaneous injection did not trigger any acute toxic effect at doses of up to 2 mg per mouse (∼0.1 g kg^–1^). Overall, this Mo_6_ compound constitutes a promising photo- and radiosensitizer for the treatment of prostatic cancer. Further experiments are planned to increase its specificity toward cancer cells by using the remaining unreacted azido groups for the grafting of aptamer/peptide that targets preferentially receptors overexpressed at the surface of prostatic cancer cells.

## Supporting Information Available

NMR characterizations of bis-dPEG11-DBCO and compound **1**, dynamic light scattering results including size distribution by number and by intensity and zeta potential of **1** in PBS, phosphorescence spectra and decay kinetics at 700 nm of fresh and 8 days old **1**, confocal microscopy images of TRAMP-C2 cells after treatment with **1**, effects of **1** in vivo including count of mice treated with **1**, spleen immune populations of mice injected with **1**, and photographs of mice, molybdenum content in tissues determined by ICP–MS 8 days after injection of the indicated amount of **1**, and additional details for in vivo toxicity studies, flow cytometry, and ICP–MS (PDF)
