# Supplementary material for: PEGylated Molybdenum–Iodine Nanocluster as a Promising Radiodynamic Agent against Prostatic Adenocarcinoma
Source: Inorg Chem. 2024 Feb 16;63(9):4419–28. doi: 10.1021/acs.inorgchem.4c00084 (PMC10915794; doi:10.1021/acs.inorgchem.4c00084)
Supplement: Supplementary file 1 — ic4c00084_si_001.pdf [file ic4c00084_si_001.pdf]

PEGylated Molybdenum-Iodine Nanocluster as a Promising Radiodynamic Agent against Prostatic Adenocarcinoma

Tomáš Přibyl,[a] Michaela Rumlová,[b] Romana Mikyšková,[c] Milan Reiniš,[c] Antonín Kaňa,[d] Karel Škoch,[e] Jaroslav Zelenka,[a] Kaplan Kirakci,[e*] Tomáš Ruml,[a*] Kamil Lang[e]

[a] Department of Biochemistry and Microbiology, University of Chemistry and Technology Prague, 166 28 Praha 6, Czech Republic

[b] Department of Biotechnology, University of Chemistry and Technology Prague, 166 28 Praha, Czech Republic

[c] Institute of Molecular Genetics of the Czech Academy of Sciences, Vídeňská 1084, 142 20 Praha, Czech Republic.

[d] Department of Analytical Chemistry, University of Chemistry and Technology Prague, 166 28 Praha, Czech Republic

[e] Institute of Inorganic Chemistry of the Czech Academy of Sciences, Řež 1001, 250 68 Husinec-Řež, Czech Republic

Content

NMR characterizations of the linker.

Figure S1. NMR characterizations of Bis-dPEG$_{11}$-DBCO and compound **1**.

Figure S2. Size distribution by number and by intensity and zeta potential of **1** in PBS, as obtained by dynamic light scattering.

Figure S3. Phosphorescence spectra and decay kinetics at 700 nm of fresh and 8-days old **1** in argon-saturated PBS.

Figure S4. Confocal microscopy images of TRAMP-C2 cells after treatment with **1**.

Table S1. Effects of **1** *in vivo*: blood count of mice treated with 0.1-2 mg of **1**.

Table S2. Effects of **1** *in vivo*: Spleen immune populations of mice injected with **1**.

Figure S5. Effects of **1** *in vivo*: Photographs of mice.

Table S3. Molybdenum content (ng/g wet weight) in tissues determined by ICP-MS 8 days after injection of the indicated amount of **1**.

Details on *in vivo* toxicity studies

NMR characterizations of the linker

NMR characterizations of Bis-dPEG$_{11}$-DBCO. For detailed assignment based on gCOSY, HMBC and HSQC experiments see Figures S1A-D.

^{1}H NMR (d_6-dmso, 600 MHz, 293 K) δ: [1.41, 1.53, 1.69, 1.82, 1.82, 2.19][each m, 1H, $(CH_2)_3$], [3.08 (dt, $^3J_{HH} \approx {}^3J_{HH} \approx$ 5.9 Hz, 2H), 3.29 (t, $^3J_{HH}$ = 5.9 Hz, 2H), 3.48 (m, overlap)]$(CH_2CH_2O)_n$, (3.60, 5.03)(each d, $^2J_{HH}$ = 14.1 Hz, 1H,CH_2N), (7.29, 7.34, 7.38, 7.62)(each m, 1H, $\underline{C_6H_4}CH_2$), (7.45, 7.45, 7.49, 7.55)(each m, 1H, $\underline{C_6H_4}N$), 7.65 (t, $^3J_{HH}$ = 5.9 Hz, 1H, CONH).

^{13}C{^{1}H} NMR (d_6-dmso, 151 MHz, 293 K) δ: (21.1, 33.4, 34.3)[$(CH_2)_3$], [38.4, 69.1, 69.5, 69.8 (overlap)][$(CH_2CH_2O)_n$], 54.8 (CH_2N), [108.2, 114.3](C≡C), [121.4 (C^{ipso}), 122.5 (C^{ipso}), 125.2 (CH), 126.8 (CH), 127.7 (CH), 128.0 (CH), 128.1 (CH), 128.9 (CH), 129.5 (CH), 132.4 (CH), 148.5 (C^{ipso}), 151.7 (C^{ipso})](2xC_6H_4), (171.5, 171.6)(2xCO).

NMR characterizations of compound **1**. Obtained material contained approximately 7% of the unreacted DBCO fragment (estimation based on the integration of ^{1}H NMR spectra). Peak assignments are based on gCOSY, HMBC and HSQC experiments, analogously with Bis-dPEG$_{11}$-DBCO experiments. For detailed assignment based on gCOSY, HMBC and HSQC experiments see Figures S1E-H.

^{1}H NMR (d_6-dmso, 600 MHz, 293 K) δ: [1.27, 1.52, 1.52, 1.70, 1.76, 1.85][each br, 1H, $(CH_2)_3$], [2.96 (br, 2H), 3.17 (br, 2H), 3.44 (m, overlap)]$(CH_2CH_2O)_n$, (4.32, 5.76)(each br d, $^2J_{HH} \approx$ 13 Hz, 1H, CH_2N), (7.06-7.68)(m, 8H, CH^{Ar}), 7.56 (br, 1H, CONH based on gCOSY).

^{13}C{^{1}H} NMR (d_6-dmso, 151 MHz, 293 K) δ: (20.6, 32.2, 34.4)[$(CH_2)_3$], [38.4, 69.1, 69.4, 69.8 (overlap)][$(CH_2CH_2O)_n$], 52.7 (CH_2N), (126.3, 126.5, 127.4, 128.1, 128.4, 128.9, 129.8, 130.2, 133.0, 133.3, 133.9, 140.5, 141.26, 143.3)(CH^{Ar}, C^{Ar} and $C^{triazole}$), (171.0, 171.7)(2xCO).

Figure S1A. **^{1}H NMR** (600 MHz, d_6-dmso, 293K) spectrum of Bis-dPEG$_{11}$-DBCO.

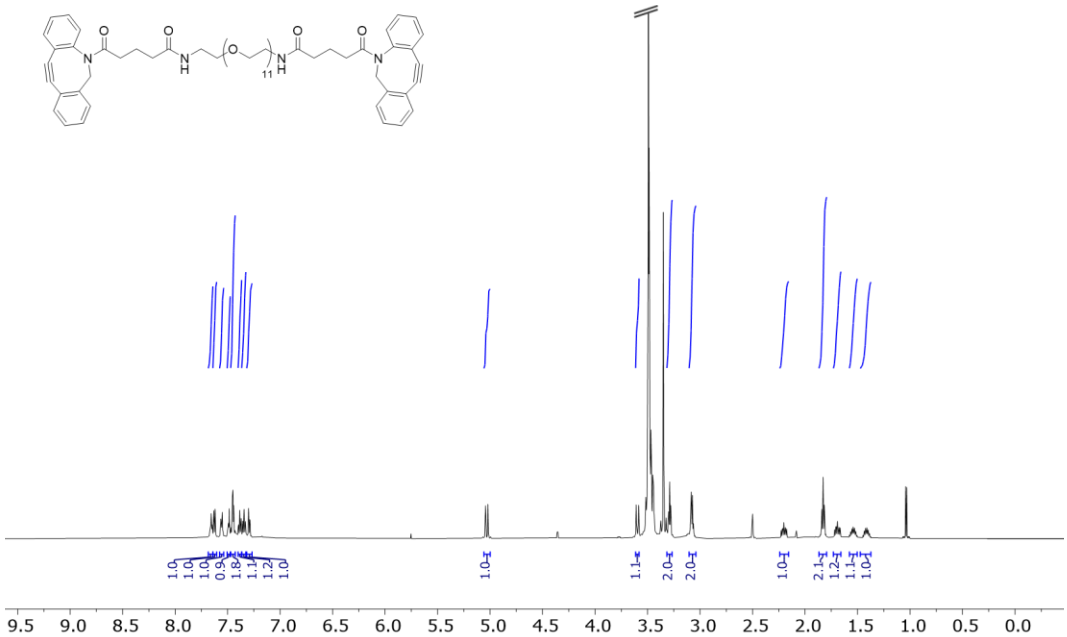

Figure S1B. Detailed view on **^{1}H NMR** (600 MHz, d_6-dmso, 293K) spectrum of Bis-dPEG$_{11}$-DBCO.

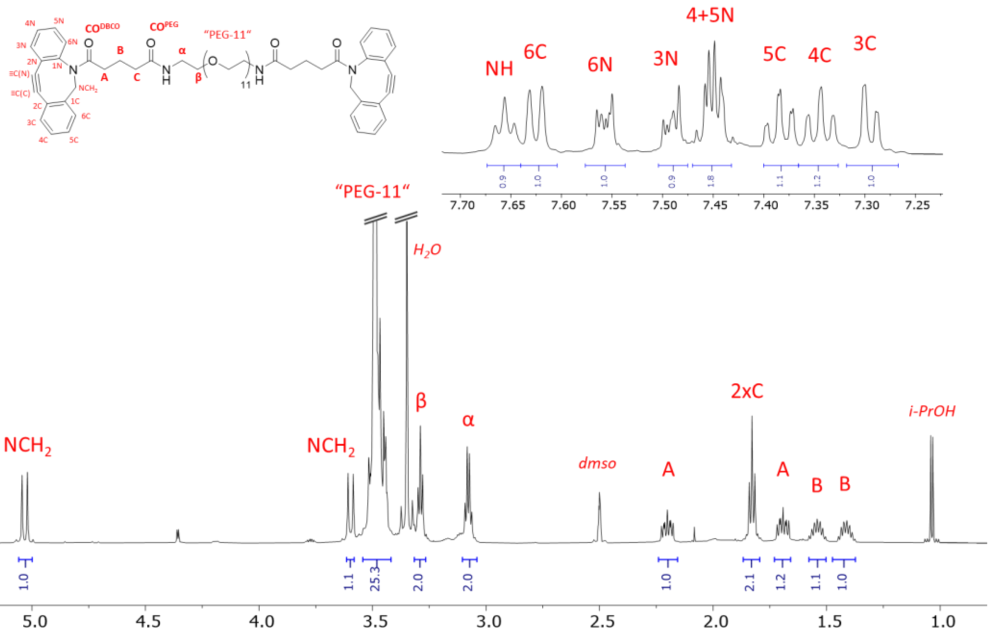

Figure S1C. **$^{13}C\{^{1}H\}$ NMR** (151 MHz, d_6-dmso, 293K) spectrum of Bis-dPEG$_{11}$-DBCO.

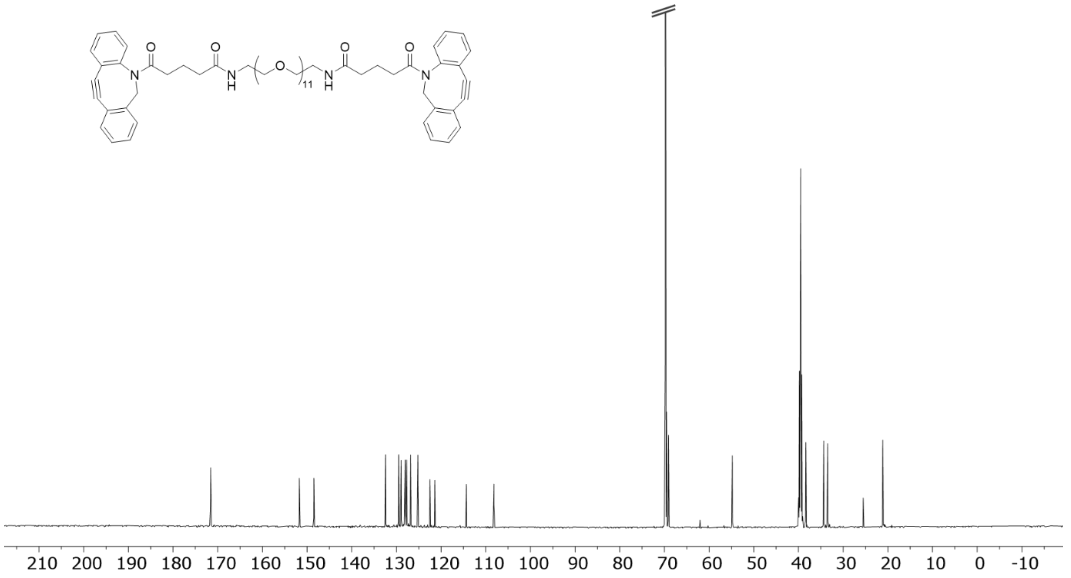

Figure S1D. Detailed view on **$^{13}C\{^{1}H\}$ NMR** (151 MHz, d_6-dmso, 293K) spectrum of compound Bis-dPEG$_{11}$-DBCO.

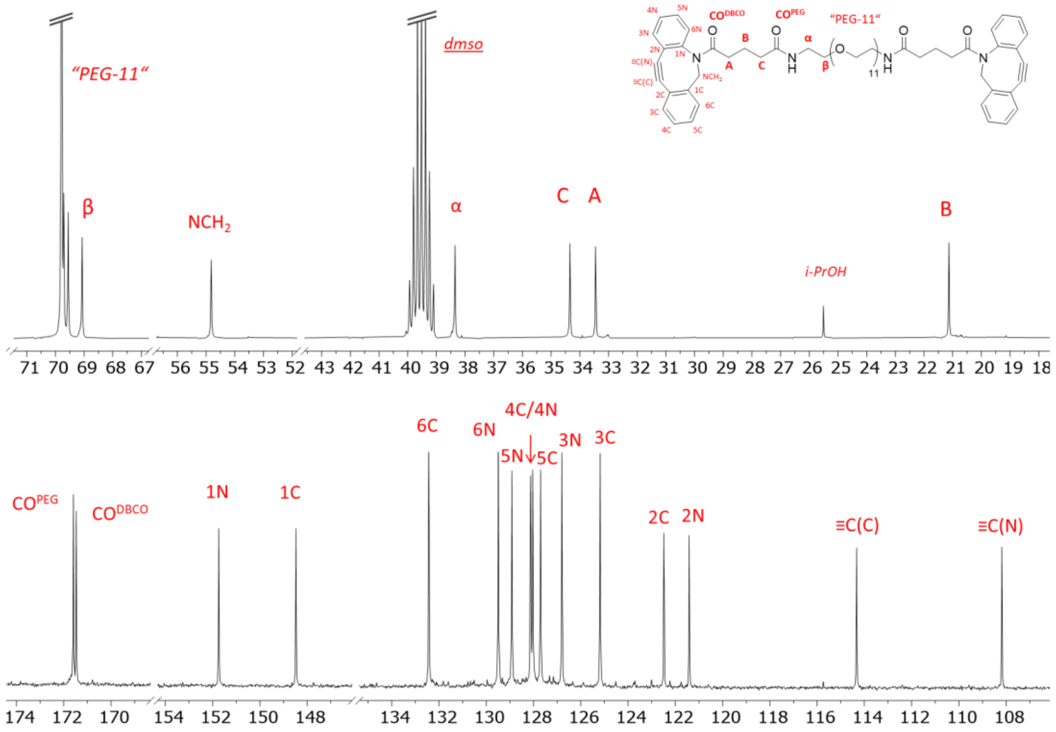

Figure S1E. **^{1}H NMR** (600 MHz, d_6-dmso, 293K) spectrum of compound **1**.

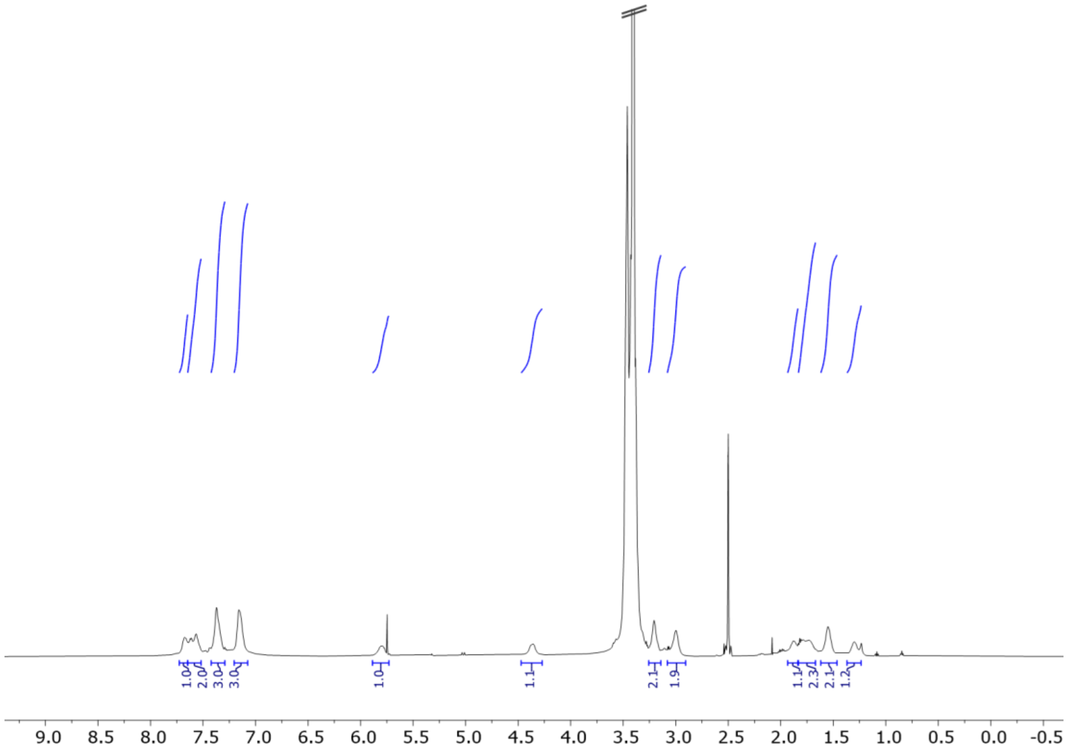

Figure S1F. Detailed view on **^{1}H NMR** (600 MHz, d_6-dmso, 293K) spectrum of compound **1**. (NOTE: # denotes signals of unreacted DBCO fragment from Bis-dPEG$_{11}$-DBCO, * denotes signal of residual dichloromethane).

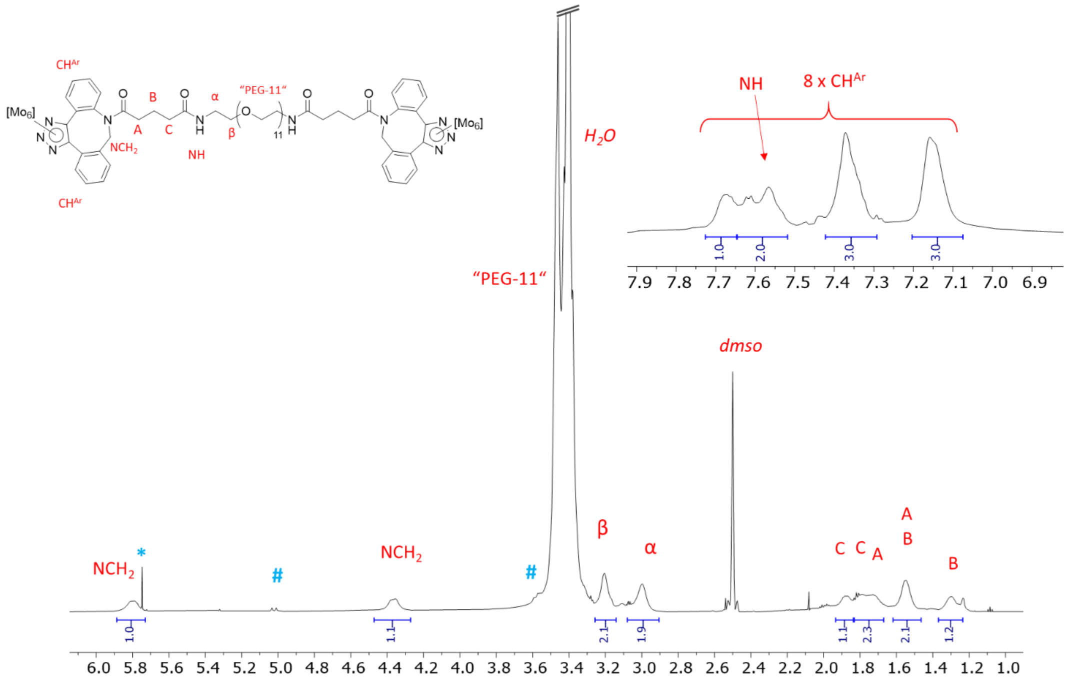

Figure S1G. **$^{13}C\{^{1}H\}$ NMR** (151 MHz, *d*$_6$-dmso, 293K) spectrum of compound **1**.

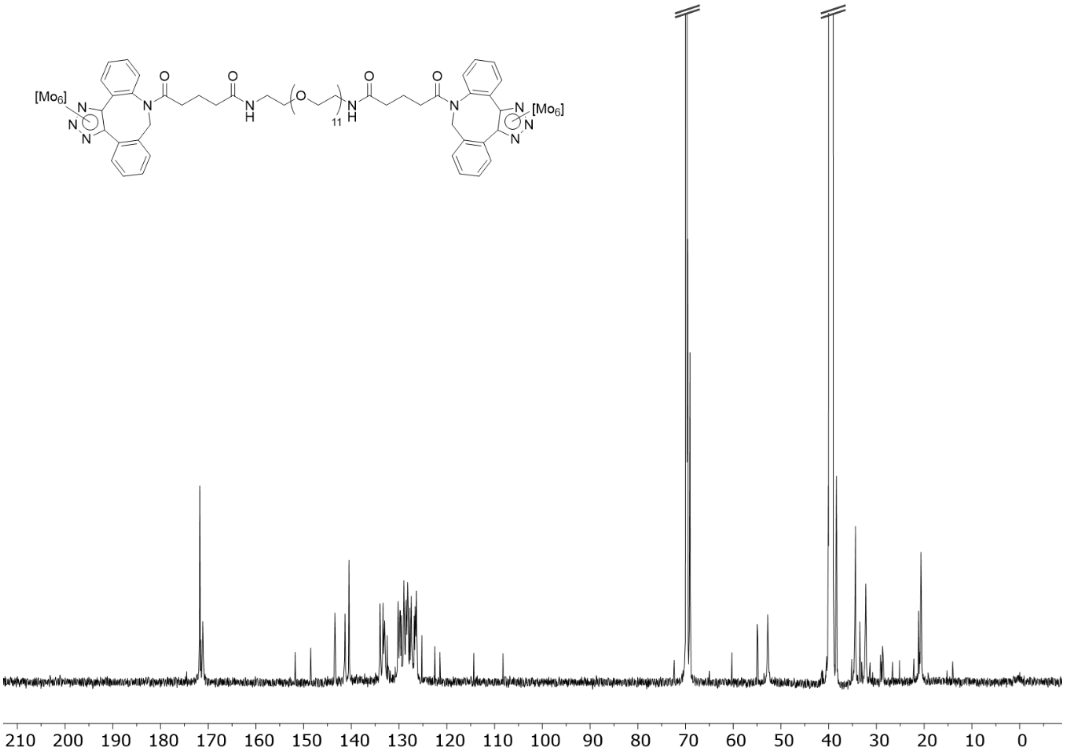

Figure S1H. Detailed view on **$^{13}C\{^{1}H\}$ NMR** (151 MHz, *d*$_6$-dmso, 293K) spectrum of compound **1**, (NOTE: # denotes residual signals of unreacted DBCO fragment).

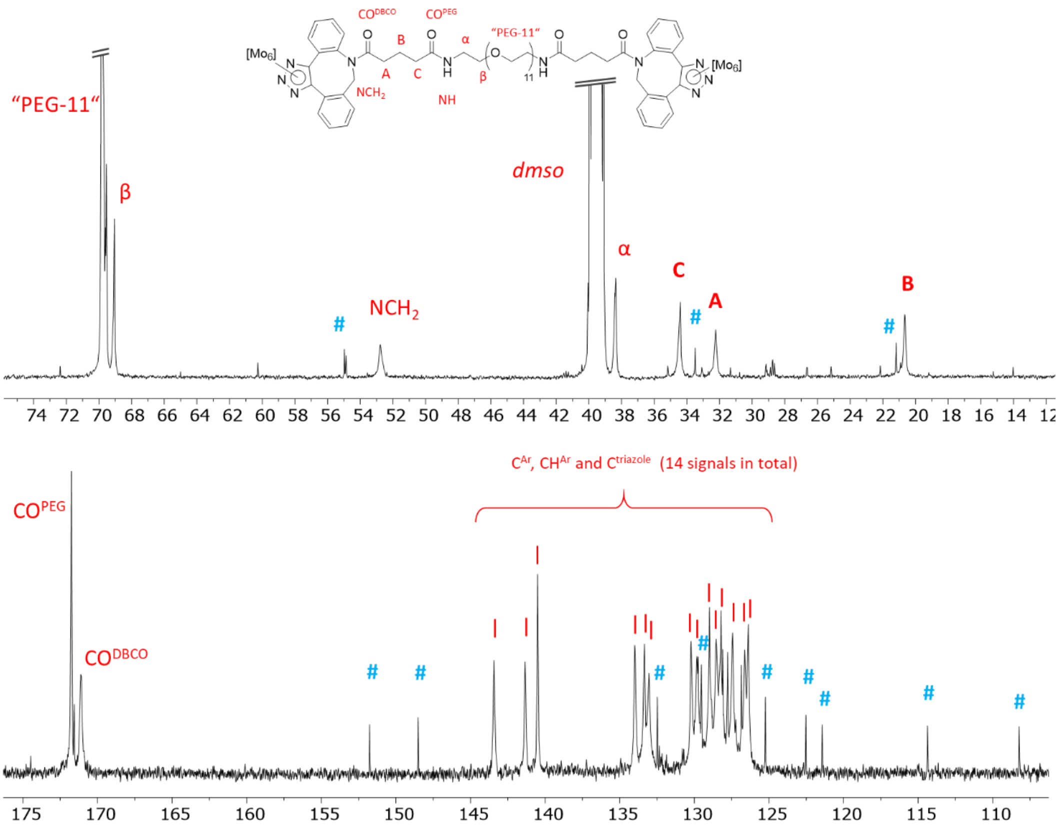

Figure S2. Size distributions by intensity and by number and zeta potentials of fresh (red) and 8-days old (green) PBS dispersions of **1**, as obtained by dynamic light scattering.

Sample	Number mean d / nm	Z-average d / nm	PDI	Zeta potential / mV
**1**, Fresh	168 ± 54	204	0.14	-8.9 ± 2.3
**1**, 8 days in PBS	189 ± 60	208	0.16	-13.4 ± 1.8

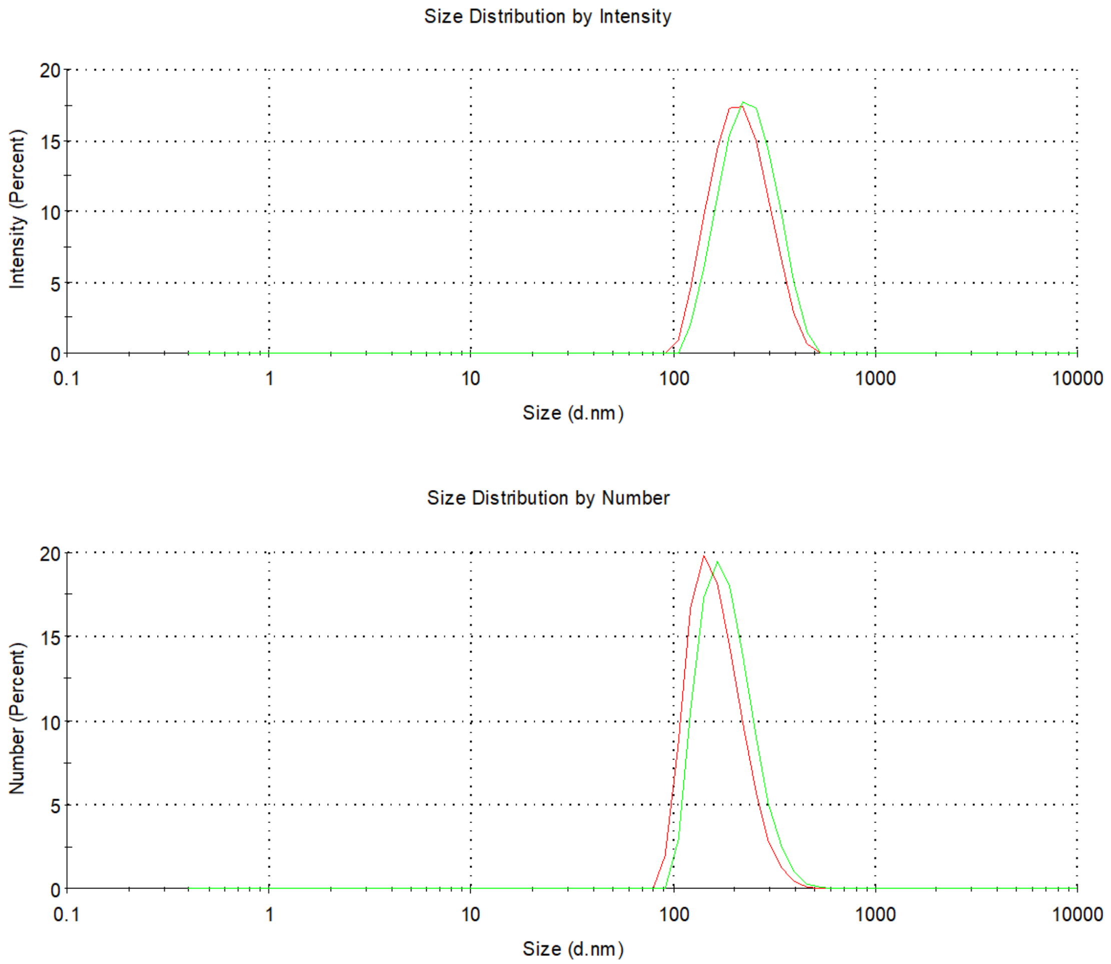

Figure S3. (A) Phosphorescence spectra of fresh (black) and 8-days old (red) **1** in argon-saturated PBS, excited at 400 nm. (B) Phosphorescence decay kinetics at 700 nm of fresh (black) and 8-days old (red) **1** in argon-saturated PBS, excited at 405 nm.

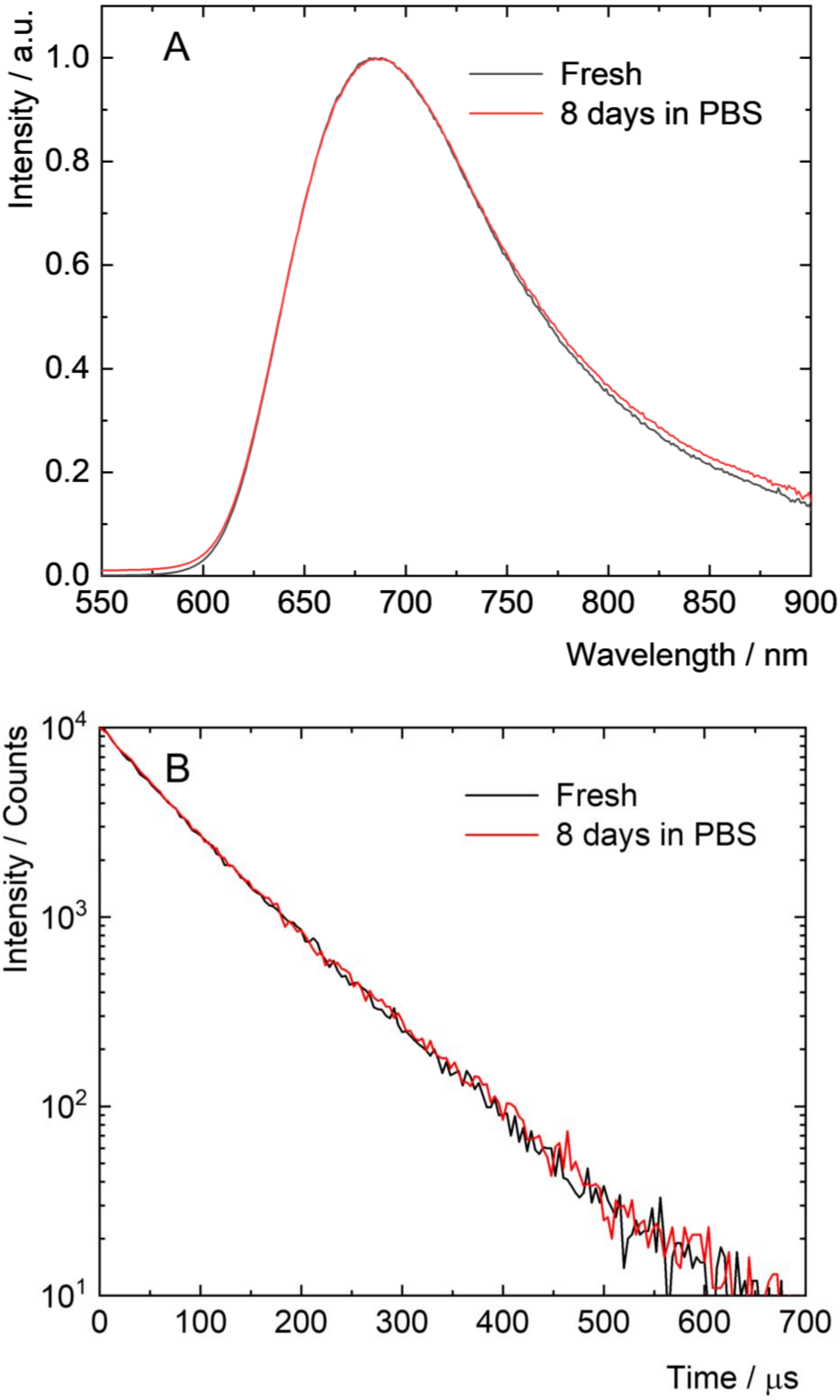

Figure S4. Confocal microscopy images of TRAMP-C2 cells after treatment with **1** and being kept in the dark for 4 hours (A) or after illumination (B). Cells were stained with DAPI (nucleus, cyan) and TMRE (mitochondrial network, magenta), Nile Red (lipids, red (membrane lipids) merged yellow (lipid droplets)) or with LysoTracker (lysosomes, yellow).

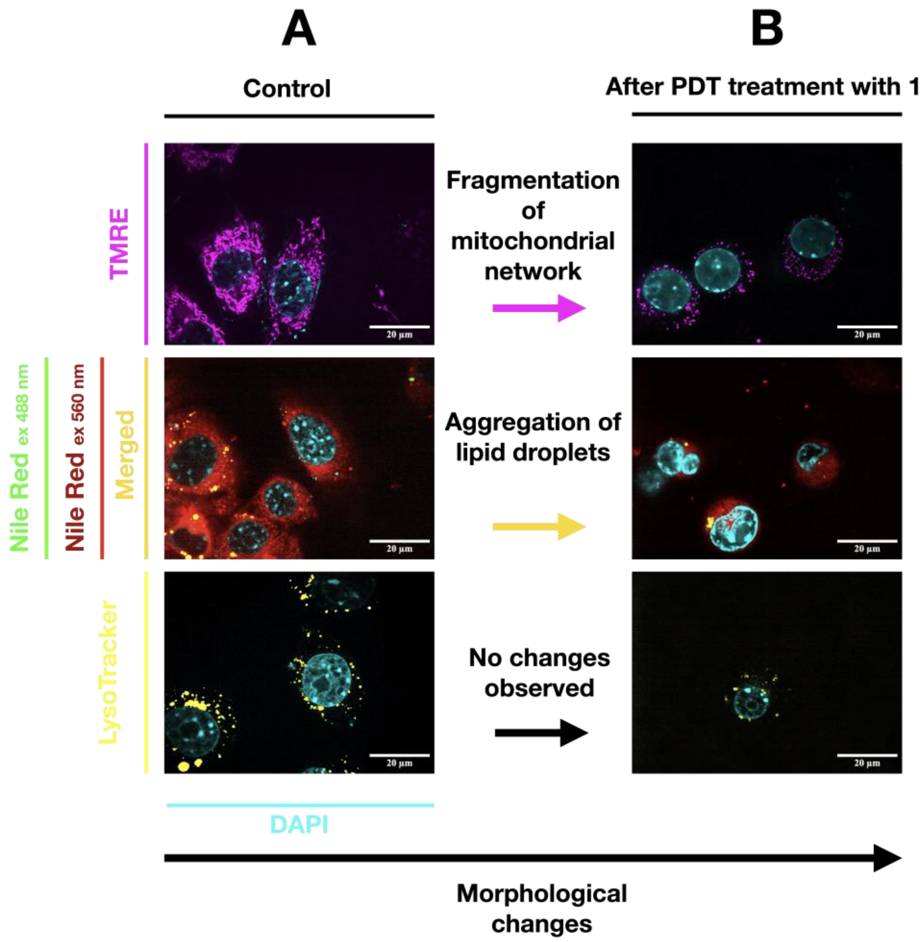

Table S1. Effects of **1** *in vivo*: blood count of mice treated with 0.1-2 mg of **1**. WBC - White blood cell count, RBC - Red blood cell count, HGB - Hemoglobin, HCT - Hematocrit, MCV - Mean cell volume, MCH - Mean corpuscular hemoglobin, MCHC - Mean cell hemoglobin concentration, RDW-CV - Red blood cell distribution width, RDW-SD – Red blood cell distribution width SD, PLT - Platelet count, MPV - Mean platelet volume, PDW - Platelet distribution width, PCT - Procalcitonin. 3 mice per group were analysed.

		WBC	RBC	HGB	HCT	MCV	MCH	MCHC	RDW-CV	RDW-SD	PLT	MPV	PDW	PCT
		x 10^9/L	x 10^{12}/L	g/L	%	fL	pg	g/L	%	fL	x 10^9/L	fL	%	mL/L
Control	AVG	4.56	11.03	171.33	0.59	53.03	15.50	292.00	0.13	28.17	927.00	5.50	14.90	5.09
	SD	1.05	2.05	34.96	0.13	1.63	0.36	6.24	0.00	1.44	65.28	0.00	0.26	0.37
2 mg	AVG	5.00	10.53	165.33	0.55	52.37	15.70	299.67	0.13	28.33	887.33	5.50	14.80	4.82
	SD	1.00	0.42	5.51	0.01	0.96	0.20	4.73	0.00	1.17	285.37	0.26	0.26	1.40
1 mg	AVG	5.27	9.75	151.67	0.51	52.20	15.60	298.33	0.13	28.00	929.33	5.50	14.70	5.07
	SD	2.09	1.06	14.64	0.06	1.04	0.26	4.93	0.00	1.11	282.45	0.17	0.00	1.41
0.5 mg	AVG	4.44	11.20	167.67	0.57	50.77	14.97	295.33	0.14	29.23	884.67	5.53	14.70	4.90
	SD	0.72	0.96	10.41	0.04	1.89	0.76	3.79	0.03	4.05	127.06	0.12	0.20	0.77
0.25 mg	AVG	4.19	9.82	155.00	0.51	51.80	15.80	304.67	0.13	27.77	876.33	5.37	14.67	4.73
	SD	1.84	0.24	5.57	0.01	0.61	0.20	4.93	0.01	1.39	53.20	0.25	0.21	0.17
0.1 mg	AVG	4.40	10.31	160.67	0.52	50.80	15.60	307.00	0.13	26.53	747.67	5.53	14.80	4.13
	SD	0.85	0.27	5.86	0.01	0.53	0.20	3.00	0.00	1.07	100.04	0.40	0.26	0.33

Table S2. Effects of **1** *in vivo*: spleen immune populations of mice injected with **1** (percentages of the CD45-positive cells are given). Splenocytes were analysed by flow cytometry for percentage of important selected immune cell populations: CD45, CD4, CD8, Gr-1+/CD11b+ and activated CD8+ immune cells (CD69+) 8 days after injection, pool of 3 mice per group.

	$CD4^+$	$CD8^+$	$Gr\text{-}1^+/CD11b^+$	$CD69^+$
Control	22.58	14.00	1.7	17.88
2 mg	22.31	14.88	0.91	15.29
1 mg	22.36	13.09	0.78	16.59
0.5 mg	21.25	11.12	0.85	24.40
0.25 mg	23.07	13.46	0.79	15.55
0.1 mg	21.22	11.38	1.45	22.73

Figure S5. Photographs of mice. The site of injection is exposed to present the yellow precipitate of **1** under the skin.

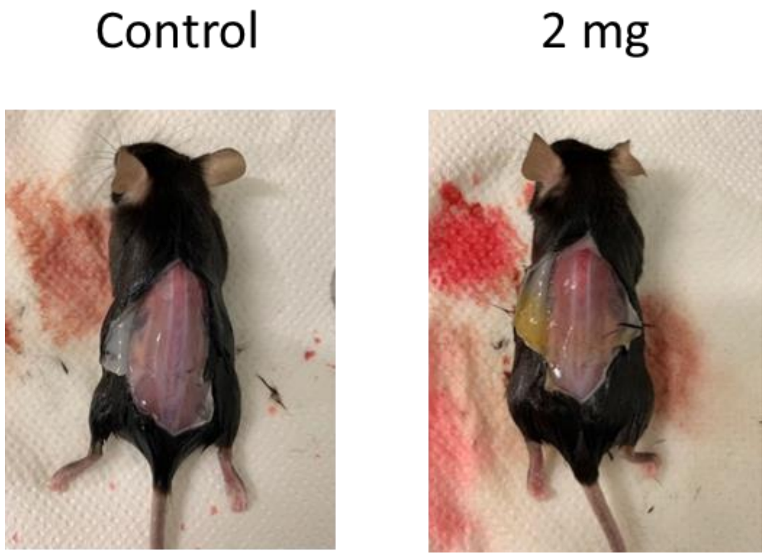

Table S3. Molybdenum content (ng g^{-1} wet weight) in tissues determined by ICP-MS 8 days after injection of the indicated amount of **1**.

		LUNG	SPLEEN	LIVER	INJECTION	KIDNEY	GALLBLADDER
Control	AVG	66	260	1170	35	430	750
	SD	3	70	110	6	16	320
2 mg	AVG	1500	4700	20000	80000	45000	5700
	SD	500	1900	8000	20000	10000	300
1 mg	AVG	930	2600	12000	45000	28000	4200
	SD	110	360	2400	12000	2400	2200
0.5 mg	AVG	580	1700	8200	24000	21000	5200
	SD	110	260	1500	6600	2800	2300
0.25 mg	AVG	230	640	3000	7300	8500	2720
	SD	40	40	270	4100	720	80
0.1 mg	AVG	150	420	2100	4300	4000	760
	SD	20	20	270	1100	480	480

*AVG, average; SD, standard deviation

In Vivo Toxicity Study

To analyse toxic effects of **1**, C57BL/6 (B6), male mice (6 mice per group) were injected subcutaneously with different doses of **1** in the range of 0.1 – 2 mg per mouse. One group was only injected with physiological saline solution and left as a control group. Mice were observed daily for signs of toxicity, and their weights were recorded. Eight days after injection, mice were anaesthetized and thereafter autopsied. Blood (25 μL) from anesthetised mice was withdrawn using a capillary pipette containing anticoagulant (heparin) from the retroorbital sinus into EDTA-containing tubes. Basic haematological parameters were measured using a Mindray BC-5300 Vet. Abdominal organs (spleen, liver, kidney) were removed for visual examination and imaging by a digital camera. Splenocytes were analysed by flow cytometry for basic immunological cell populations (CD4+, CD8+, CD11b+/Gr-1+, CD69+). Single-cell suspensions were prepared by spleen homogenization through a cell strainer (70 mm; BD Biosciences, San Jose, CA, USA). Erythrocytes were osmotically lysed using ammonium chloride–potassium lysis buffer, and the cell suspension was washed three times in the RPMI-1640 medium and utilised. Selected organs (skin - site of injection, kidney, gallbladder, spleen, lung, liver) were subsequently frozen in liquid nitrogen to analyse Mo concentration in tissue using ICP-MS.

Flow cytometry

The expression of cell surface molecules on spleen cells was analysed by flow cytometry. Cell suspensions were washed and preincubated with anti-CD16/CD32 (2.4G2) antibody to minimize non-specific binding for 15 min at 4˚C following the washing step and incubation with labelled primary antibody for 30 min at 4˚C. Relevant isotype controls of irrelevant specificity were used. The FACS buffer (PBS, 1% FBS, 0.1% NaN3) was used for all washing steps and analysis. The following antibodies were used for FACS analyses: BD: BV421 anti-mouse CD45 (30-F11), PE anti-mouse CD8a (53-6.7), BV711 anti-mouse CD4 (RM4-5), FITC anti-mouse CD69 (H1.2F3), APC anti-mouse Ly-6g/Ly-6C (Gr-1) (RB6-8C5), and BV711 anti-mouse CD11b (M1/70)APC-CD45 (30-F11). FACS analysis was performed using a flow cytometer Symphony (BD Biosciences) and analysed using the FlowJo 10 software (FlowJo LLC, Ashland, OR, USA).

Inductively coupled plasma mass spectrometry

The samples (whole organs or aliquot of homogenized organs) for measurement of the total amount of molybdenum were weighed into a Teflon® vessel, and 3 mL of concentrated 65% nitric acid (high-purity Analpure®, Analytika, Praha, Czech Republic) was added. The digestion procedure was performed using a microwave-heated digestion system Speedwave 4 (Berghof, Eningen unter Achalm, Germany). The temperature was ramped in 8 min to 200 °C and maintained at that temperature for 10 min. The digest was quantitatively transferred into a 50 mL volumetric flask, internal standard indium (Astasol® 1000 ± 2 mg L^{-1}, Analytika, Praha, Czech Republic) was added in an amount corresponding to a final concentration of 20 µg L^{-1}, and the flask was filled to mark with water (Milli-Q, Millipore, Bedford, MA, USA). Molybdenum concentrations were measured by inductively coupled plasma mass spectrometry (ICP-MS, PerkinElmer, Concord, ON, Canada). Quantification was carried out via external calibration. The ICP-MS measurement conditions were as follows: RF power 1.1 kW, nebulizer gas flow rate 0.76 L min^{-1}, auxiliary gas flow rate 1 L min^{-1}, plasma gas flow rate 11 L min^{-1}, measured isotope ^{98}Mo as an analyte and ^{115}In as an internal standard.
